# Extracellular Vesicles: Classification, Biological Functions, Diseases, and Therapeutic Opportunities

**DOI:** 10.1002/mco2.70896

**Published:** 2026-09-20

**Authors:** Sabrina Garbo, Francesco Marocco, Clemens Zwergel, Marco Tripodi, Cecilia Battistelli

**Affiliations:** ^1^ Department of Molecular Medicine Sapienza University of Rome Rome Italy; ^2^ Department of Drug Chemistry and Technologies Sapienza University of Rome Rome Italy

## Abstract

Extracellular vesicles (EVs) are lipid bilayer‐enclosed particles released by virtually all cell types and act as mediators of intercellular communication in physiology and disease. By transporting diverse bioactive molecules, including proteins, lipids, and nucleic acids, EVs regulate gene expression, modulate immune responses, and support tissue homeostasis. Rapid advances have revealed their heterogeneity, complex biogenesis, and broad functional relevance, as well as their importance as biomarkers and therapeutic delivery systems. The molecular principles governing selective cargo loading remain incompletely understood, particularly for microRNAs: once viewed as passive carriers, EVs are now known to sort miRNAs through regulated mechanisms that do not simply reflect intracellular abundance. In this review, we provide an integrated overview of EV biology, including classification, biogenesis, and roles in health and disease. We focus on mechanisms underlying selective RNA cargo loading, highlighting RNA‐binding proteins such as SYNCRIP and PCBP2, sequence features linked to selective export, and epitranscriptomic regulation, including m6A modifications. We also discuss emerging computational and AI‐driven approaches to identify RNA–protein interactions and determinants of cargo selection, alongside developments in EV engineering and clinical translation. By bridging mechanistic insights with translational perspectives, this review positions EVs as platforms for precision medicine, biomarker discovery, and RNA‐based therapeutics.

## Graphical abstract

• EV content maintains cellular homeostasis and is involved in several disease onset and progression.

• miRNA export in EVs depends on: RNA‐binding proteins recognizing motif sequences and on epitranscriptomic modifications.

• The disclosure of delivery mechanisms is crucial for developing personalized EV‐based therapeutic tools.

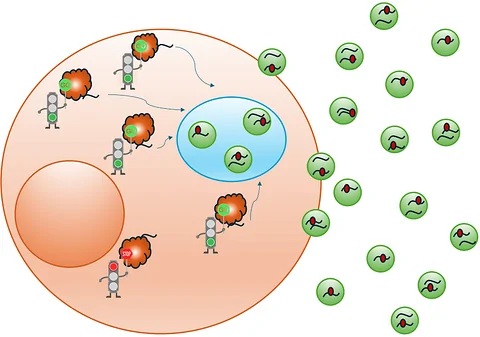

## 1 Introduction

Extracellular vesicles (EVs) have represented a major focus of research over the past few decades. They are structurally diverse, and their significance became more widely recognized when their roles in diagnosing and treating various diseases were explored [1]. In particular, the use of EVs as diagnostic tools has grown rapidly, becoming a valuable source of information when analyzed via liquid biopsies, a common method for detecting abnormal disease‐related marker expression in pregnant women, children, and other at‐risk groups. Furthermore, this approach enables early detection of biomarkers due to its minimally invasive nature [2]. Analyzing EV cargo has identified potential new biomarkers associated with neurological diseases, cardiac issues, immunological disorders, and cancer. The translational value of dissecting EV molecular cargo with regard to disease onset and progression has fuelled curiosity about the molecular mechanisms behind EV‐cargo loading [3, 4]. This pursuit serves to both understand the basic biological processes and to develop strategies to direct specific molecules into EVs for therapeutic purposes. Pioneering studies have revealed the central roles of RNA‐binding proteins (RBPs), sequence determinants [5, 6, 7, 8] and RNA modifications such as epitranscriptomic modifications [9]. Indeed, the direct recognition of specific consensus sequences by RBPs can be influenced by post‐transcriptional modifications of RNA molecules, which in turn can promote or impair the export of molecular cargo into EVs. In this context, elucidating the fundamental mechanisms that control EV‐cargo loading is crucial not only to fill a knowledge gap in the field of cell‐to‐cell communication and disease pathophysiology but also to lay the groundwork for developing EV‐based therapies. Understanding the endogenous mechanisms that govern the selective loading of cargo into EVs has stimulated efforts to apply these principles to biotechnology and therapeutic strategies—aiming to restore the tissue‐ and organ‐specific plethora of molecules encapsulated in vesicles that play central roles in intercellular communication.

Understanding the historical foundations and evolution of EV research is essential for contextualizing current advances and shaping future directions. EVs are lipid bilayer‐enclosed particles secreted by virtually all cell types and are now widely recognized as critical mediators of intercellular communication. Once considered inert cellular debris, EVs have undergone a significant conceptual re‐evaluation over the past decades, emerging as key regulators of both physiological and pathological processes. The earliest evidence of EV‐like structures dates back to 1967, when Wolf described “platelet dust” released from activated platelets as byproducts of cellular activation or damage [10]. This perception persisted until the 1980s, when studies on reticulocyte maturation revealed a regulated vesicular secretion pathway involving multivesicular bodies (MVBs) and the release of small vesicles, later termed exosomes [11, 12]. These findings marked the first indication that vesicle release is a biologically controlled process, not just a passive phenomenon. A major paradigm shift occurred in the 1990s with the discovery that EVs play a role in immune regulation. The discovery that EVs participate in immune regulation through the transfer of functional major histocompatibility complex (MHC) molecules [13], together with the later finding that they transport mRNAs and miRNAs capable of altering gene expression in recipient cells, established EVs as active signaling entities and vehicles of intercellular genetic exchange [14]. This discovery induced widespread interest across multiple disciplines such as oncology, neuroscience, cardiovascular biology, and regenerative medicine. Advances in imaging, omics technologies, and isolation methods have further revealed the remarkable heterogeneity of EV populations [15, 16]. Additionally, the presence of EVs in virtually all biofluids has also underscored their potential as minimally invasive biomarkers and platforms for therapeutic delivery [17].

EVs are classified based on their biogenesis, size, and molecular composition [15, 16] and are enriched in bioactive cargos that collectively reflect the cellular state and microenvironment of their parent cells. EV biogenesis is a highly regulated process; exosomes originate from the endosomal pathway through the formation of MVBs and their subsequent fusion with the plasma membrane, a mechanism regulated by the endosomal sorting complex required for transport (ESCRT) machinery as well as by ESCRT‐independent pathways [18]. In contrast, microvesicles are generated by outward budding of the plasma membrane, involving cytoskeletal remodeling and changes in membrane lipid asymmetry. These distinct biogenetic routes contribute to the compositional and functional diversity of EV populations. Once released, EVs mediate intercellular communication through multiple mechanisms, including receptor–ligand interactions, membrane fusion, and endocytic uptake by recipient cells. Through these processes, EVs influence various biological functions such as immune modulation, angiogenesis, tissue repair, and metabolic regulation [17]. Importantly, EV‐mediated signaling allows for spatial and temporal coordination of communication, especially within complex tissue microenvironments. The presence of EVs in virtually all biological fluids—including blood, urine, saliva, cerebrospinal fluid, and breast milk—has made them attractive candidates for noninvasive biomarker discovery [19]. Additionally, their inherent stability, biocompatibility, and capacity to cross biological barriers highlight their potential as natural delivery vehicles for therapeutic agents.

Over the past decade, EV research has shifted from descriptive characterization to mechanistic and translational studies. High‐throughput proteomics, lipidomics, and transcriptomics have enabled comprehensive, though still ongoing profiling of EV cargo, revealing disease‐specific signatures (e.g., associated with cancer, neurodegenerative disorders, cardiovascular diseases [CVDs], and inflammatory conditions) [20]. As discussed in more detail below, in oncology, EVs are now recognized as critical mediators of tumor‐microenvironment crosstalk. Tumor‐derived EVs promote angiogenesis, immune evasion, extracellular matrix (ECM) remodeling and metastatic niche formation, while stromal cell‐derived EVs reciprocally influence tumor progression and therapy resistance [21, 22]. In parallel, increasing attention has been devoted to the therapeutic potential of EVs. Mesenchymal stem/stromal cell‐derived EVs have shown regenerative and immunomodulatory effects in preclinical models of tissue injury and inflammatory diseases, often mimicking the benefits of cell‐based therapies while reducing safety concerns [23, 24]. Furthermore, engineered EVs are being developed as drug delivery platforms, capable of transporting small molecules, proteins, and nucleic acids with increased specificity and reduced immunogenicity [25, 26, 27, 28, 29, 30, 31, 32, 33]. Despite these advances, the absence of standardized methods for isolation, quantification, and characterization limits reproducibility and cross‐study comparability. In response, international efforts such as the Minimal Information for Studies of Extracellular Vesicles (MISEV) guidelines have provided a framework to improve methodological rigor and reporting standards [19, 34]. Furthermore, unresolved questions about EV heterogeneity, biodistribution, and functional specificity remain active areas of research.

Collectively, the current state of the art reflects a rapidly evolving field in which technological innovation and conceptual refinement are advancing EVs toward clinical use. Continued integration of mechanistic insights with standardized methods will be crucial to unlock the diagnostic and therapeutic potential of EVs fully.

This review provides an integrated overview of EV biology, with particular emphasis on the molecular mechanisms governing selective RNA cargo loading. To guide the reader through the field, the review is structured as follows: we first discuss EV classification, heterogeneity, isolation, and characterization, followed by their physiological and pathological roles. We then examine the mechanisms regulating selective RNA sorting, including the contribution of RBPs, sequence motifs and epitranscriptomic modifications. Finally, we highlight emerging computational and artificial intelligence (AI)‐based approaches, therapeutic applications, clinical translation, and future directions in the EV field.

## 2 EV Heterogeneity, Isolation, and Characterization

### 2.1 Classification Based on Biogenesis, Size, and Cargo

EVs constitute a heterogeneous family of membrane‑bound particles released by virtually all cell types and conserved across evolution, from bacteria to mammals. Their diversity reflects differences in biogenesis, size, molecular composition, and functional specialization, making EV classification a central challenge in the field.

Historically, EVs have been grouped into three major categories: apoptotic bodies, microvesicles, and exosomes [35]. Apoptotic bodies (500–5000 nm) are generated during apoptosis; they encapsulate genomic DNA fragments, histones, RNAs, proteins, and cytosolic components, and are typically cleared by phagocytes [36].

Microvesicles (100–1000 nm) are produced by outward budding of the plasma membrane. In cancer, large microvesicles (oncosomes) mediate the transfer of oncogenic cargo and support tumor–stroma communication [37].

**FIGURE 1:**
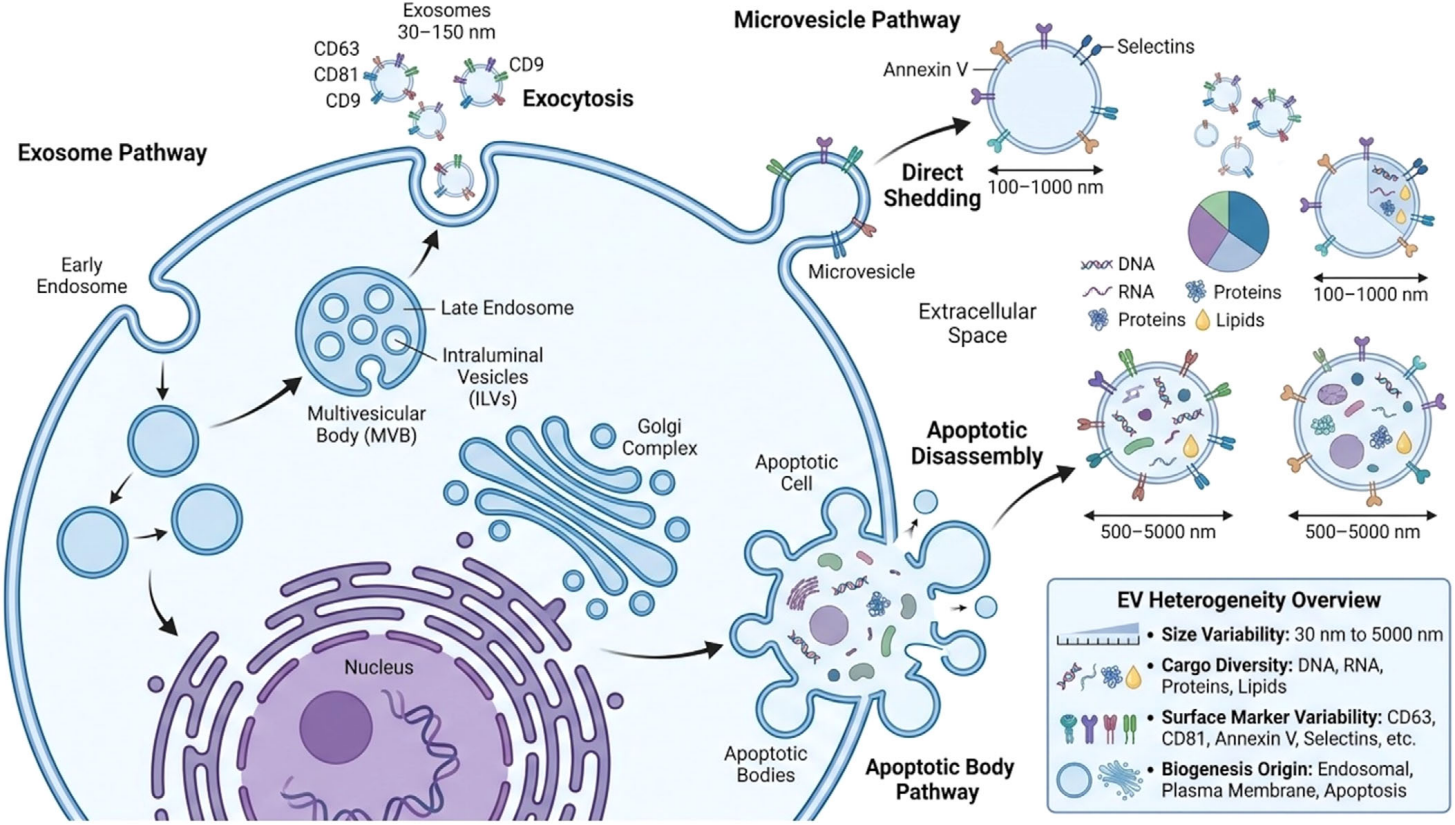
Comprehensive diagram illustrating the biogenesis, secretion, and heterogeneity of EVs. Schematic representation of heterogeneous EV populations released through three main routes: exosomes generated via the endosomal system, microvesicles shed directly from the plasma membrane, and apoptotic bodies produced during programmed cell death. The EV heterogeneity overview panel highlights differences in size, cargo composition (DNA, RNA, proteins, and lipids reflecting the physiological or pathological state of the originating cell), and surface markers, which influence EV detectability and functional impact. Clinically, these features underpin the use of EVs as minimally invasive biomarkers, potential therapeutic carriers, and modulators of disease processes such as inflammation, fibrosis, and cancer progression. This figure was created with FigureLabs (www.figurelabs.ai).

Exosomes are between 30 and 150 nm in diameter and originate from the endosomal membrane. After budding, vesicles are released as intraluminal vesicles (ILVs) into the lumen of MVBs, and then they are secreted upon the fusion of MVBs with the plasma membrane [38] (Figure 1).

Since EV subtypes can be challenging to identify based solely on biochemical features, the latest classification guidelines recommend describing them as “small” or “large” EVs [19, 34]. EVs are characterized by the presence of general and specific molecules on their surface that reflect their origin, localization, and secretion mechanisms [39]. This characterization is important not only for determining the origin of EVs but also because these molecules can serve as biomarkers specific to certain pathophysiological conditions (such as CD24 and EpCAM in ovarian cancer EVs, PCA3 in prostate cancer EVs, EGFRvIII in glioblastoma multiforme EVs, HER‐2 in breast cancer EVs).

More generally, the combination of expression of tetraspanins CD9, CD63, and CD81 is typical of exosomes, while CD9, CD63, and CD82 characterize microvesicles. Calnexin, a component of the endoplasmic reticulum (ER), is considered a negative marker for exosomes. In contrast, Alix and Tsg101 are more abundant on the surface of exosomes, as they are part of the endosomal trafficking machinery [39].

Furthermore, EVs can also display on their surface MHC Class I and II, as well as fusion and transport proteins (e.g., GTPases, flotillin, Rab2, Rab7, Rab11, annexins), metabolic enzymes (e.g., GAPDH, enolase 1, PKM2, PGK1), and signal transduction molecules (e.g., protein kinases, 14‐3‐3, G proteins) [39, 40].

Concerning the intraluminal cargo, EVs contain proteins, coding, and noncoding RNAs [41, 42], nuclear DNA fragments [43, 44], and mitochondrial DNA fragments [45], which depend on the cell of origin and the pathophysiological conditions, enabling EVs to modulate gene expression, metabolism, immune responses, and tumor progression (Figure 1).

EVs can also be classified based on their cell type of origin. These classifications include platelet‐derived EVs, oncosomes from cancer cells and prostasomes from prostate cells [46]. Recent research has also introduced new classifications and names, such as migrasomes, stress EVs, exomeres, matrix vesicles, autophagic EVs, and nonvesicular particles [46].

Migrasomes are vesicles released by retraction fibers during migration, playing a crucial role in cell‐to‐cell communication, embryonic development and tumorigenesis [47]. Stress EVs are vesicles released in response to stress conditions and cancer therapeutics [48]. Exomeres are nonmembranous vesicles rich in proteins and enzymes involved in various metabolic pathways [49]. Matrix vesicles are mainly involved in initiating bone and cartilage calcification [50]. Autophagic EVs are released under autophagic conditions and have a functional role in viral infection dynamics [51, 52]. The classification of EVs (Table 1) is an ongoing process, and scientists continue to consider various biological and molecular criteria in their research.

**TABLE 1:** Classification and comparison of the characteristics of major EV subtypes.

EV subtype	Biogenesis	Size range	Surface markers	Cargo profile	Functional/ Biological notes	References
Apoptotic bodies	Cell fragmentation during apoptosis	500–5000 nm	Histones, Annexin V, phosphatidylserine	Genomic DNA, RNAs, proteins, organelles	Cleared by phagocytes; carry large DNA fragments; reflect apoptotic disassembly	Colombo et al., 2014 [15] Thery et al., 2002 [16] Van Niel et al., 2022 [35]
Microvesicles	Outward budding of the plasma membrane	100–1000 nm	CD9, CD63, CD82, Annexins, Integrins	Cytosolic proteins, RNAs, lipids, mitochondrial fragments	In cancer, large microvesicles (oncosomes) transfer oncogenic cargo and mediate tumor–stroma communication	Colombo et al., 2014 [15] Thery et al., 2002 [16] Van Niel et al., 2022 [35]
Exosomes	ILVs formed in MVBs and released by fusion with plasma membrane	30–150 nm	CD9, CD63, CD81, ALIX, TSG101	miRNAs, mRNAs, proteins, lipids, metabolites	Central mediators of intercellular communication; enriched in regulatory RNAs	Colombo et al., 2014 [15] Thery et al., 2002 [16] Van Niel et al., 2022 [35] Gurung et al., 2021 [38] Van Niel et al., 2018 [39]
Large oncosomes	Shedding from highly migratory or transformed cancer cells	1–10 µm	ARF6, Cav 1	Oncogenic proteins, DNA, metabolic enzymes	Tumor‐specific EVs carrying aggressive signaling cues	Tricarico et al., 2017 [37]
Exomeres	Nonmembranous nanoparticle; unclear biogenesis	∼35 nm	Marker poor	Metabolic enzymes, nucleic acids	Distinct biodistribution; metabolic specialization	Anand et al., 2021 [49]
Migrasomes	Released from retraction fibers during cell migration	200–3000 nm	TSPAN4	Proteins, RNAs	Roles in development, tissue patterning, tumorigenesis	Zhang et al., 2023 [47]
Stress EVs	Released under stress or therapeutic pressure	Variable	Stress‐induced markers	Stress‐response proteins, RNAs	Reflect cellular stress states; relevant in cancer therapy	Eguchi et al., 2020 [48]
Matrix vesicles	Budding from chondrocytes/osteoblasts	100–300 nm	ALP, Annexins	Mineralization enzymes, Ca^2^^+^/PO_4_^3^^−^	Initiate bone and cartilage calcification	Anderson et al., 2003 [50]
Autophagic EVs	Released during autophagy‐related secretion	Variable	LC3‐associated markers	Autophagy‐related proteins, RNAs	Implicated in viral infection dynamics and stress adaptation	Mao et al., 2025 [51] Mao et al., 2026 [52]
Small EVs	Operational size‐based category	< 200 nm	Tetraspanins, ESCRT proteins	RNAs, proteins, lipids	Recommended nomenclature when biogenesis cannot be assigned	MISEV 2023 [53]
Large EVs	Operational size‐based category	> 200 nm	Plasma membrane markers	Proteins, DNA, organelles	Useful when biochemical markers are insufficient	MISEV 2023 [53]

Abbreviations: ALP, alkaline phosphatase; ARF6, ADP‐ribosylation factor 6; Cav1, caveolin‐1; ESCRT, endosomal sorting complex required for transport; ILVs, intraluminal vesicles; LC3, microtubule‐associated protein 1 light chain 3; MVBs, multivesicular bodies; TSPAN4, tetraspanin‐4.

### 2.2 Current Methods for EV Isolation and Purification: Strengths and Limitations

The isolation of EVs remains one of the most persistent technical challenges in the field. This difficulty stems from the intrinsic heterogeneity of EV populations and the substantial overlap in size, density, and biochemical composition among different vesicle subtypes. As a result, no single method can universally guarantee high purity, high yield, and preservation of EV integrity. Instead, it is required to carefully select the most appropriate strategy based on the biological question, sample type and downstream applications [54].

Among the available approaches, differential ultracentrifugation remains the most widely used. It is considered the gold standard for bulk EV isolation because it is inexpensive and applicable to large sample volumes. However, it is also labor‐intensive, time‑consuming, and prone to coisolating contaminants such as protein aggregates and lipoproteins, which can confound functional assays [55].

To improve purity, a second approach employs density‑gradient centrifugation. By separating particles according to density, this method enriches for bona fide EVs and reduces contamination. Nevertheless, it is technically demanding, offers limited scalability, and may not be suitable for high‑throughput workflows [56].

Size‑exclusion chromatography (SEC) has gained popularity because it is reproducible and preserves vesicle morphology and functionality. SEC effectively removes soluble proteins, but it may dilute EV samples and coelute particles of similar size, such as lipoproteins or protein complexes [57].

Ultrafiltration provides a rapid and scalable alternative, particularly useful for concentrating EVs. However, interactions with filter membranes can lead to vesicle deformation, loss, or selective retention of specific EV subpopulations [58].

More selective approaches rely on immunoaffinity capture, which uses antibodies against surface markers such as CD63, CD9, or EpCAM to isolate defined EV subsets. This strategy offers high specificity but typically yields low quantities and depends heavily on the availability and reliability of surface markers [59].

Finally, microfluidic platforms represent an emerging class of technologies that enable rapid, low‑volume, and high‑precision EV isolation. These systems integrate size‑based, immunoaffinity, or physical separation into miniaturized devices. While promising, their adoption remains limited by cost, lack of standardization, and variable throughput [60].

Table 2 summarizes these different methods.

**TABLE 2:** Comparison of EV isolation and purification methods.

Method	Principle of separation	Advantages	Limitations/biases	Best suited for	References
Differential ultracentrifugation	Sequential centrifugation steps based on sedimentation rate	Inexpensive; scalable to large volumes; widely adopted “gold standard”	Labor‐intensive; time‐consuming; coisolates protein aggregates and lipoproteins; variable purity	Bulk EV isolation; general EV studies; large‐volume biofluids	Kim et al., 2025 [55].
Density‐gradient centrifugation	Separation based on buoyant density	High purity; reduces contaminants; enriches bona fide EVs	Technically demanding; low scalability; not ideal for high‐throughput workflows	High‐purity preparations; proteomics; functional assays requiring clean EVs	Brennan et al., 2020 [56].
Size‐exclusion chromatography (SEC)	Separation by size through porous matrices	Gentle; preserves morphology and functionality; reproducible; removes soluble proteins	Dilutes samples; coelutes similarly sized particles (lipoproteins, protein complexes)	Functional assays; RNA profiling; clinical‐grade EV preparations	Sidhom et al., 2020 [57]
Ultrafiltration	Membrane‐based size retention	Rapid; scalable; useful for concentration	Vesicle deformation; loss or selective retention; membrane interactions introduce bias	Preconcentration; workflows requiring speed; combination with other methods	Kronilov et al., 2018 [58]
Immunoaffinity capture	Antibody‐based capture of surface markers (e.g., CD63, CD9, EpCAM)	High specificity: isolates defined EV subpopulations	Low yield; dependent on marker availability and reliability; expensive	Subtype‐specific studies; biomarker discovery; tumor‐derived EV enrichment	Khanabdali et al., 2024 [59]
Microfluidic platforms	Miniaturized devices integrating size‐based, immunoaffinity, or physical separation	Rapid; low‐volume; high precision; multiplexing potential	Costly; limited standardization; variable throughput; emerging technology	Single‐EV analysis; point‐of‐care applications; small sample volumes	Dai et al., 2025 [60]

Abbreviation: EpCAM, epithelial cell adhesion molecule.

Overall, no single method provides complete purity, and each approach introduces specific biases in terms of EV subtype recovery. For this reason, the choice of isolation strategy must be guided by the intended downstream analysis (e.g., proteomics, RNA profiling, functional assays, or single‑particle characterization) and, ideally, complemented by orthogonal validation techniques.

### 2.3 Single‑EV Analysis and the Challenge of Heterogeneity

A major limitation of traditional EV research is that most analytical approaches rely on bulk measurements, which inevitably mask the remarkable heterogeneity that characterizes EV populations. EVs released even from a single cell type can differ substantially in size, biogenesis, surface composition, and molecular cargo, leading to functional diversity that cannot be captured by general assays. This realization has driven the rapid development of single‑particle technologies, which are now reshaping our understanding of EV biology.

One of the most widely adopted tools is high‑resolution flow cytometry [61], which has evolved to detect vesicles below 200 nm when combined with optimized optics and fluorescent labeling strategies. Although still limited by sensitivity and background noise, this approach enables multiparametric analysis of individual vesicles and is increasingly used to profile EV surface markers at the single‑particle level.

Nanoparticle tracking analysis (NTA) remains a cornerstone for determining EV size distribution and concentration [62]. While NTA lacks molecular specificity, its ability to track the Brownian motion of individual particles provides valuable information on EV heterogeneity within a sample, especially when combined with fluorescence‑based detection.

Techniques such as atomic force microscopy and electron microscopy offer unparalleled ultrastructural resolution, revealing differences in vesicle morphology, membrane stiffness, and surface topology. Their low throughput, however, limits their use to detailed characterization rather than routine profiling.

More recently, Raman spectroscopy [63] and mass spectrometry‑based single‑EV proteomics have emerged as powerful tools capable of generating molecular fingerprints of individual vesicles. These methods can distinguish EV subpopulations based on their biochemical composition, providing insights into selective cargo loading and disease‑associated signatures.

A particularly transformative development is the advent of droplet‑based and barcoded single‑EV‐RNA profiling platforms [64]. By encapsulating individual vesicles into microdroplets or assigning unique molecular barcodes, these technologies enable high‑throughput quantification of RNA cargo at single‑vesicle resolution. This has revealed striking variability in miRNA and mRNA content, supporting the concept that EV secretion is a selective and regulated process, rather than a passive release of cellular debris.

Collectively, these single‑EV approaches demonstrate that vesicles differ not only in size and biogenesis, but also in cargo composition, surface marker expression, and functional potential. This complexity underscores the need for refined classification systems and highlights why bulk analyses alone are insufficient to fully capture the biological roles of EVs.

### 2.4 Toward Standardization: MISEV2023 Guidelines

As the EV field continues to expand, the need for rigorous standardization has become increasingly evident. Variability in isolation procedures, characterization strategies, and reporting practices has historically limited the reproducibility and comparability of EV studies across laboratories. To address these challenges, the International Society for Extracellular Vesicles (ISEV) released the MISEV2023 guidelines [53], which provide an updated and comprehensive framework for conducting and reporting EV research.

A central principle of MISEV2023 is the rigorous documentation of isolation methods. Researchers are encouraged to describe each step of their workflow in detail, including centrifugation parameters, filtration steps, buffer composition, and any enrichment or depletion strategies. This level of transparency is essential for enabling reproducibility and for interpreting differences between studies that may arise from methodological variation.

The guidelines also emphasize the importance of transparent, multiparametric EV characterization. Rather than relying on a single marker or technique, MISEV2023 recommends using multiple complementary approaches (e.g., protein markers, particle quantification, imaging, and functional assays) to confirm EV identity.

The adoption of orthogonal validation strategies (combining biochemical markers with imaging, density profiling, or functional readouts) helps ensure that isolated particles are indeed EVs and not contaminants such as lipoproteins, protein aggregates, or cell debris.

MISEV2023 further highlights the need for standardized functional assays, urging researchers to define experimental conditions, normalization strategies, and controls with precision. This is particularly important for studies assessing EV uptake, signaling, or therapeutic potential, where methodological inconsistencies can lead to divergent conclusions.

Importantly, the guidelines explicitly recognize the growing relevance of single‑EV technologies, multiomics approaches, and reference materials. These innovations are seen as essential tools for addressing EV heterogeneity and for harmonizing data across platforms and laboratories. By integrating these advanced methodologies into standardized workflows, the field can move toward more robust, comparable, and biologically meaningful EV analyses.

Overall, MISEV2023 represents a major step toward methodological rigor, providing a shared foundation that will support the maturation of EV research and its translation into clinical and technological applications.

## 3 Physiological Functions of EVs

**FIGURE 2:**
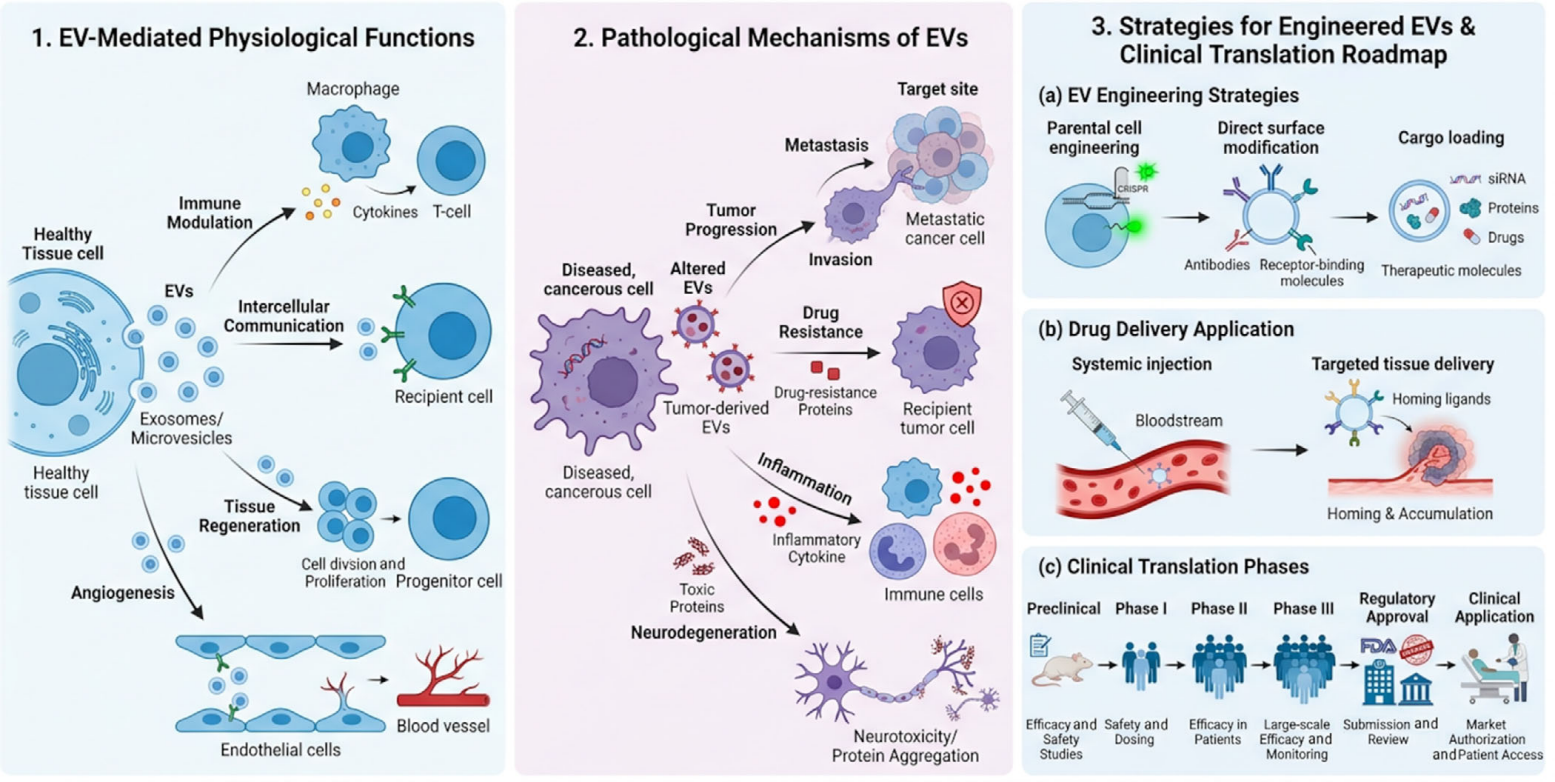
Overview of EV‐mediated physiological functions, pathological mechanisms, and clinical approaches/translation roadmap. Left: Schematic representation of EVs supporting key physiological processes (including immune modulation, tissue repair, and angiogenesis) by mediating targeted communication between stromal, immune, and endothelial cells. Middle: Schematic representation of EVs contribution to tumor progression, drug resistance, inflammation, and neurodegeneration through the transfer of pathogenic proteins and nucleic acids. Right: Schematic representation of the emerging therapeutic opportunities offered by engineered EVs. Strategies for surface modification, cargo loading, and targeted delivery (Panels a and b), alongside a defined roadmap for clinical translation from preclinical validation to regulatory approval (Panel c), are indicated. This figure was created with FigureLabs (www.figurelabs.ai).

EVs are increasingly recognized as mediators of intercellular communication through the transfer of their specific informational cargo of molecules. This allows EVs to influence gene expression and cell behavior in recipient cells. As a result, EVs are considered signalosomes for various key biological processes in pathophysiology [65]. Regarding their role in physiological processes, EVs have been shown to maintain homeostasis, regulate the immune system, facilitate various neurological functions and contribute to cellular metabolism (Figure 2, left panel).

### 3.1 Immune Regulation and Intercellular Communication

EVs may activate immune responses or suppress inflammation in a tolerogenic manner, contributing to immune surveillance. EVs confer immunosuppression through various mechanisms, such as enhancing the function of regulatory T cells, suppressing natural killer (NK) and CD8+ cell activity, and inhibiting both monocyte differentiation into dendritic cells (DCs) and DC maturation. In contrast, EVs can promote immune activation by enhancing the proliferation and survival of hematopoietic stem cells and activating monocytes, B cells, and NK cells [66, 67]. It has been reported that EVs can be produced by neutrophils, monocytes and monocyte‐derived macrophages in response to inflammatory stimuli, tissue damage, or pathogen‐associated signals. Notably, they play a crucial role in restoring homeostasis and can act either directly at the site of injury or by enhancing the immune functions of their parent cells [68, 69, 70]. Furthermore, neutrophil‐derived EVs increase the expression of proinflammatory and adhesion molecules in endothelial cells (ECs), thereby facilitating leukocyte transmigration across endothelial barriers [71]. Interestingly, since EVs are enriched in chemokines, they may guide leukocytes to sites of inflammation [72]. Additional studies have demonstrated that EVs express both Class I and Class II MHC molecules, as well as adhesion and costimulatory molecules, enabling EVs to directly stimulate CD8+ and CD4+ T cells by binding to their respective plasma membrane receptors [73]. It has been further demonstrated that DCs pulsed with tumor peptides release immunogenic exosomes that induce a more robust CD8+ T‐cell antitumor response compared to T cells incubated with the peptides alone, supporting the enhanced immunogenicity theory. This EV‐associated improvement in the inflammatory response has been utilized in trials of EV‐mediated antitumor therapy. In vivo, it has been suggested that EVs exhibit higher tumor antigen density and the presence of heat shock protein (HSP), which may function as an adjuvant in activating the immune system [74]. Consequently, it has been proposed that DCs can be primed with tumor‐associated proteins, thereby inducing activation of tumor‐specific cytotoxic leukocytes as a vaccine‐related therapeutic approach.

In addition to activating the immune system, EVs possess the ability to suppress immunity, functioning in a homeostatic way. For example, the cytotoxic activity of NK‐derived exosomes was only observed in activated immune cells, not in resting cells [75]. This suppressive role of EVs is particularly significant during times when a proinflammatory state could be detrimental, such as during pregnancy. Additionally, EVs from immature DCs have been shown to be enriched with TGF‐β1 and IL‐10, which block T‐cell proliferation [76]. As a result, EV‐mediated immunosuppression is vital for preventing autoimmunity and chronic inflammation.

### 3.2 Cardiovascular Homeostasis

EVs can be released by various cardiac cell types and play a vital role in intercellular communication. They influence pathways impacting angiogenesis, apoptosis, inflammation, proliferation, and fibrosis. Studies have shown that cardiomyocytes (CMs), cardiac fibroblasts (CFs), and ECs release EVs with distinct miRNA content, capable of modulating the behavior of other cardiac cells [77]. Moreover, it has been proposed that they maintain homeostasis and contribute to vascular calcification, a significant factor in CVDs [78]; EVs are also involved in postplaque rupture responses, such as thrombotic vessel occlusion [79]. Furthermore, in the case of CVDs, EVs have potential as diagnostic tools and therapeutic agents. However, challenges in isolating and characterizing EVs, along with the lack of appropriate experimental models, hinder a comprehensive understanding of their role.

The living myocardial slice (LMS) model, which preserves the structural and electrophysiological features of adult cardiac tissue, offers a valuable platform for studying EV dynamics and their impact. Indeed, the LMS model, an ultra‐thin layer of myocardium, provides a more physiologically relevant and feasible approach to study EVs compared to conventional cell culture and animal models [80]. Specifically, it preserves the multicellular and ECM components of adult cardiac tissue, facilitating investigation within a biomimetic environment.

In blood circulation, EVs participate in the coagulation cascade by providing a surface for the assembly of clotting factors [81]. It has been reported that platelet EVs consist of proteins derived from the plasma membrane, cytosol, and organelles, including adhesion receptors, coagulation factors, transcription factors, growth factors, active enzymes, cytokines, and chemokines [82, 83]. Additionally, various proteins act as key mediators of cell‐to‐cell interactions, such as GP IIb/IIIa (CD41/CD61), GP Ib (CD42b), P‐selectin (CD62P), and CD40L (CD154). Platelet EVs also carry markers once considered exclusive to exosomes, such as CD9, CD63, CD81, HSP70, and TSG101. The cargo within platelet EVs is complex, showing both pro‐ and anticoagulant functions [84, 85]. Moreover, they contain small metabolites and RNAs, including protein‐coding mRNAs, miRNAs, YRNAs, and circular RNAs, originating from parent megakaryocytes [86].

It has also been reported that EVs contribute to blood pressure regulation by being enriched with angiotensin II type I receptors [87]. Circulating EVs isolated from normotensive rats and humans have demonstrated the ability to prevent acetylcholine‐induced vasodilation ex vivo. This inhibition was attributed to EV‐mediated suppression of eNOS, suggesting that EVs may regulate nitric oxide production [88].

### 3.3 Central Nervous System Physiology

The brain contains the most diverse cell types, with dynamic and complex structures, making it challenging to find cell‐specific and rare markers in the central nervous system (CNS). Nevertheless, tissue dissociation techniques can isolate EVs directly from the brain [89]. Within the CNS, EVs are released from various cell types, including neurons, astrocytes, microglia, oligodendrocytes, and vascular cells. Efficient, nonpathological neurotransmission depends on intercellular communication, and increased neuronal activity is associated with increased EV release, which is vital for synapse maintenance. This neuronal activity‐dependent role of EVs is especially important during development, particularly during activity‐dependent pruning of synapses. Intercellular communication also plays a key role in regional CNS development. Sonic hedgehog (Shh) signaling guides cortical development, and liquid flow around the developing mouse embryo releases EVs carrying Shh, contributing to lateralization. Studies in chick embryos show that Shh is present in EVs, and that different EV subpopulations express molecules that regulate Shh signaling through accessory molecule expression [90]. These in vivo studies provide crucial evidence of the importance of EVs during normal CNS development.

In the adult CNS, essential intercellular interactions continue to maintain normal function. Microglia need to stay quiescent, the blood–brain barrier (BBB) must remain intact, and astrocytes need to stay healthy. Although direct evidence for EV roles in these processes is limited, insights from other fields suggest their potential involvement. For instance, CX3CL1 (fractalkine) on neurons binds to CX3CR1 on microglia, promoting quiescence [91]. Fractalkine, released from apoptotic neurons, signals injury, facilitating debris clearance after trauma [92]. ECs release fractalkine on their surface, attracting CX3CR1+ monocytes as guidance cues [93].

The BBB consists of pericytes, astrocytes, and ECs. Tight junctions in ECs prevent cell migration and large‐molecule entry, unlike the fenestrated vasculature of the peripheral circulation. Early studies showed the ability of EVs to cross the BBB [94]. More recently, EVs have been found to travel between the periphery and brain [95] and between the brain and periphery [96], effectively enabling communication across the intact BBB. While this offers therapeutic promise, the complex intercellular communication within the neurovascular unit requires careful consideration. For instance, brain pericytes produce proangiogenic EVs, suggesting a role in regulating BBB growth and function [97].

Recently, EVs have been recognized as key mediators of communication within the gut–brain axis. They act as nanoscale carriers of bioactive molecules, enabling bidirectional signaling between the gut, the immune system and the CNS. EVs are released by intestinal epithelial cells, immune cells, neurons of the enteric nervous system and gut microbiota—including bacteria that secrete outer membrane vesicles capable of influencing host physiology [98]. Their cargo can modulate intestinal barrier integrity, immune activation and neuroinflammatory pathways. Notably, microbiota‐derived EVs have been shown to cross the intestinal epithelium and, in some cases, the BBB, thereby directly affecting neuronal signaling, synaptic plasticity, and behavior [99]. Additionally, changes in EV biogenesis or cargo composition have been linked to neurological and psychiatric disorders, supporting their role as critical molecular links between gut dysbiosis and brain dysfunction, as well as their potential utility as biomarkers and therapeutic targets in gut–brain axis‐related diseases [100].

### 3.4 Stem Cell Niche Maintenance

EVs also contribute to stem cell maintenance and plasticity. They seem to play an essential role in repairing injured tissues due to their neoangiogenic, antiapoptotic, and cell‐proliferation‐stimulating properties [2, 101]. Specifically, it has been reported that EVs derived from embryonic stem cells (ESCs) promote pluripotency by activating focal adhesion kinase (FAK) through fibronectin–integrin interactions [102, 103]. This finding suggests that ESC‐derived EVs may help stabilize the pluripotent state and prevent premature differentiation during embryo development. Furthermore, the potential of ESC‐derived EVs as a cell‐free therapeutic tool to suppress the tumorigenic phenotype of cancer cells has been reported. Specifically, human embryonic stem cell‐derived EVs (hESC‐EVs) inhibit the growth of MCF‐7 and A‐375 cells in a dose‐dependent manner, while promoting the proliferation of normal cells [104, 105]. This effect is attributed to the modulation of the Nodal signaling pathway and the downregulation of pluripotency transcription factors. Additionally, hESC‐EVs induce apoptosis, lower Ki67 expression, and downregulate cell cycle and antiapoptotic genes in cancer cells. They also reduce the stemness of cancer cells, demonstrated by decreased colony and sphere formation [106]. Moreover, hESC‐EVs inhibit cancer cell migration, decrease epithelial‐to‐mesenchymal transition (EMT)‐related gene expression, and reduce tumor growth in vivo [106]. Although hESC‐EVs show significant potential as a cancer treatment, challenges remain in understanding their mechanisms of action, optimizing their application and addressing potential risks.

## 4 Pathological Roles of EVs in Human Diseases

EVs play central roles in a wide range of physiological processes. However, accumulating evidence has also highlighted their involvement in the onset and progression of diverse pathological conditions (Figure 2, middle panel).

### 4.1 Mechanisms of EV‐Mediated Pathogenesis (Inflammation, Fibrosis, Immune Evasion, Metabolic Reprogramming)

Concerning their proinflammatory and pathogenic roles, EVs can carry autoantigens that trigger autoreactive T and B cell responses. EVs originating from mesenchymal stem cells (MSCs) or DCs can deliver anti‐inflammatory molecules (such as TGF‐β, IL‐10, and miR‐146a) to target immune cells, thereby suppressing T‐cell activation and promoting regulatory T‐cell expansion [107, 108, 109]. Additionally, EVs can induce macrophage polarization toward an anti‐inflammatory M2 phenotype, thereby reducing tissue damage in models [110, 111]. EV cargo affects key signaling pathways, influencing cytokine production, antigen presentation and immune cell survival. miRNAs within EVs (e.g., miR‐21, miR‐155, miR‐146a) regulate gene expression post‐transcriptionally in recipient cells, fine‐tuning immune responses in either a pro‐ or anti‐inflammatory direction [112, 113, 114].

Mechanistically, EVs exert their functional effects by modulating key intracellular signaling pathways, including TLR, NF‑κB, JAK‑STAT, MAPK, and interferon pathways, which affect cytokine production, immune cell recruitment, apoptosis and antiviral defenses (e.g., EV‑associated miRNAs can regulate TLR/NF‑κB signaling and cytokine expression) [112]. EV cargo, especially miRNAs such as miR‑155, miR‑146a, miR‑21, and miR‑223, can post‑transcriptionally regulate host genes involved in immune activation, inflammation and pathogen sensing, providing a finely tuned mechanism for both pathogen exploitation and host defense (e.g., the transfer of miR‑155 and miR‑146a via exosomes modulates inflammatory responses to endotoxin) [112].

Moreover, the content and function of EVs can change depending on cell type, pathogen type, infection stage, and host genetic background, emphasizing their versatile and context‐specific nature.

Fibrosis is the pathological outcome of a failed wound‐healing response, characterized by the excessive accumulation of ECM proteins. EVs serve as essential “fibrogenic messengers,” facilitating the crosstalk between injured epithelial or ECs and the effector cells of fibrosis (primarily myofibroblasts). In chronic Hepatitis C, EVs released into the plasma remain enriched with profibrogenic proteins even after the virus has been cleared by therapy. These vesicles carry a specialized cargo (including a deficit of antifibrogenic miRNAs), that continuously activates HSCs, ensuring that hepatic remodeling persists despite the absence of an active viral load [115]. Following a myocardial infarction (MI), injured CMs release EVs containing stress‐related proteins and “fibromiRs” (such as miR‐21). These vesicles are internalized by CFs, triggering their transformation into myofibroblasts [116]. In inflammatory bowel disease (IBD), EVs from intestinal epithelial cells transport miRNAs and cytokines that not only recruit immune cells but also activate mesenchymal cells in the lamina propria [117]. In the vascular system, EVs influence the behavior of vascular smooth muscle cells (VSMCs). By promoting a synthetic (rather than contractile) phenotype, EVs drive the deposition of fibrous tissue within the arterial wall, which paradoxically can lead to plaque instability if the fibrous cap is degraded by EV‐associated proteases (matrix metalloproteinases [MMPs]) [118, 119]. EVs do not just signal for fibrosis; they often carry the “tools” for it. Many EVs are enriched with MMPs and their inhibitors (TIMPs). By delivering these enzymes directly to the ECM, EVs enable precise, localized degradation of healthy tissue and its replacement with disorganized collagen, fundamentally altering the mechanical properties of the organ [120].

Immune evasion mediated by EVs represents one of the most sophisticated strategies for pathological survival. By transferring immunosuppressive molecules and sequestering key components of the immune system, EVs allow viruses, bacteria, and dysfunctional cells to persist and replicate by effectively “blinding” or disarming the host's defenses. In viral infections such as HIV, SARS‐CoV‐2, and HCV, EVs often display viral surface antigens [121]. These “pseudoviruses” bind and sequester neutralizing antibodies, preventing them from reaching the actual virions and significantly reducing the efficacy of the humoral immune response. Pathogenic EVs can be enriched with complement regulatory proteins (e.g., CD55, CD59), which inhibit the formation of the membrane attack complex, thereby protecting infected cells or pathogens from lysis [122]. In HCV infection, virus‐modified EVs transport specific miRNAs that target immunoregulatory signaling pathways. Functionally, this leads to the inhibition of NK cell degranulation, stripping the host of a critical primary defense against infected cells. Pathogen‐derived EVs, such as those from *Mycobacterium tuberculosis*, deliver mycobacterial lipids and proteins that alter macrophage and DC activation [123]. This results in the downregulation of MHC‐II molecules and costimulatory signals, preventing the proper priming of T cells and fostering immune tolerance toward the infection.

A critical dimension of EV‐mediated pathogenesis is the ability of these vesicles to induce metabolic reprogramming in recipient cells. By delivering a complex cargo of enzymes, metabolic intermediates, and noncoding RNAs, EVs can “rewire” the energetic and biosynthetic pathways of target cells, shifting them from a homeostatic state to one that supports inflammation, chronic disease, or pathogen survival. In the context of CVDs, EVs are central players in the metabolic dysfunction of the arterial wall. EVs derived from inflamed ECs or activated macrophages transport inflammatory lipids and miRNAs (such as miR‐21) that regulate cholesterol efflux and lipid processing. In atherosclerosis, this leads to the pathological accumulation of intracellular lipids in macrophages, driving their transformation into foam cells [124]. The influence of EVs on metabolic reprogramming extends beyond local interactions. In chronic inflammatory states like systemic lupus erythematosus (SLE) or heart failure, circulating EVs carry metabolic “stress signals” that can induce altered lipid handling in distant organs [125, 126]. This systemic metabolic interference contributes to the multiorgan dysfunction characteristic of these pathologies, in which the EV‐mediated transfer of miRNAs acts as a fine‐tuning mechanism for host gene expression involved in nutrient sensing and energy balance [127].

### 4.2 EVs as Disease Biomarkers: From Proof‐of‐Concept to Clinical Validation

The transition of EVs from laboratory curiosities to robust clinical biomarkers represents one of the most promising frontiers in precision medicine. As “liquid biopsies,” EVs offer a noninvasive window into the physiological and pathological state of their cells of origin, shielding fragile molecular cargo (such as proteins, lipids, and various RNA species) from degradation in systemic circulation [20, 128]. Initial proof‐of‐concept studies have successfully demonstrated that EV signatures can distinguish healthy individuals from those with conditions like SLE, rheumatoid arthritis (RA), or chronic viral infections [129, 130, 131]. For example, specific miRNA profiles within plasma EVs or the presence of autoantigens in synovial fluid‐derived vesicles have shown high sensitivity in identifying early‐stage inflammation.

However, moving toward clinical validation requires overcoming significant hurdles related to standardization and scalability. Current challenges include the heterogeneity of isolation methods (e.g., ultracentrifugation vs. microfluidics), which can lead to variability in purity and yield, thus complicating the establishment of universal reference ranges. Furthermore, for an EV‐based biomarker to be clinically viable, it must demonstrate rigorous analytical validity, clinical sensitivity, and the ability to predict treatment response or disease flare‐ups in large, diverse patient cohorts. As high‐throughput technologies like nanoscale flow cytometry and advanced proteomic profiling mature, the integration of EV analysis into routine diagnostics (e.g., monitoring the “fibrogenic potential” of EVs in post‐treatment HCV patients or tracking “cytokine storms” via host EVs) is poised to revolutionize how we diagnose, stage, and manage chronic and infectious diseases.

### 4.3 Disease‐Specific Vignettes (Autoimmune/Inflammatory, Cardiovascular, Neurodegenerative, Cancer)

In the context of autoimmune and inflammatory pathologies, EVs function as dynamic rheostats of the immune system, exerting a dual role that oscillates between disease promotion and immunoregulation. Their pathogenic impact is primarily driven by the transport of autoantigens and proinflammatory mediators. For instance, in SLE, EVs enriched with nuclear antigens like DNA and histones activate plasmacytoid DCs to secrete type I interferons, which worsen systemic autoimmunity [132, 133]. In RA, EVs derived from synovial fibroblasts and immune cells carry TNF‐α, IL‐6, MMPs, and miRNAs (such as miR‐155 and miR‐146a), thereby promoting synovial inflammation, cartilage degradation and osteoclast activation [134]. In IBD, EVs from intestinal epithelial cells and immune cells modulate gut immunity by transporting proinflammatory cytokines and miRNAs, which contribute to the disruption of the mucosal barrier [135, 136].

EVs participate in both disease propagation and tissue repair mechanisms across multiple CVD phenotypes, making them central players in cardiovascular pathophysiology, as well as promising diagnostic and therapeutic tools [137]. EVs contribute to atherosclerosis, the chronic inflammatory arterial disease underlying most coronary and cerebrovascular events, by modulating endothelial function, promoting monocyte recruitment, driving foam cell formation and influencing plaque stability through the transfer of inflammatory lipids, adhesion molecules, miRNAs, and proteases. They influence lipid metabolism and VSMC behavior, exacerbating plaque development and potential rupture. In MI and ischemic heart disease, EVs released from injured CMs and other cardiac cells carry stress‐related miRNAs and proteins that interact with inflammatory cells and progenitor cells, modulating postischemic inflammatory responses, fibrosis and repair processes. Circulating EVs reflect the severity of cardiac injury and may serve as liquid biomarkers for acute cardiac events. EVs also play roles in heart failure, where maladaptive intercellular signaling from CM‐ and fibroblast‐derived EVs drives pathological remodeling, hypertrophy, interstitial fibrosis and impaired contractile function. Their cargoes (including noncoding RNAs and profibrotic factors) contribute to progressive cardiac dysfunction [138, 139].

In neurodegenerative diseases such as Alzheimer's disease (AD), Parkinson's disease (PD), amyotrophic lateral sclerosis (ALS), and Huntington's disease (HD), EVs can propagate pathogenic proteins (including amyloid‐β, tau, and α‐synuclein) facilitating the spread of neurotoxic aggregates between cells. This process contributes to disease progression, synaptic dysfunction and neuronal loss [140]. Similarly, EVs modulate microglial activation and astrocyte‐mediated neuroinflammation, which are central to the pathophysiology of these disorders [141]. Beyond their pathogenic roles, EVs circulating in the peripheral blood and cerebrospinal fluid serve as minimally invasive indicators of CNS pathology. Small EVs (sEVs), enriched with disease‐associated miRNAs and proteins, have demonstrated potential as biomarkers for early detection, disease classification and monitoring therapeutic responses, especially in AD and other cognitive disorders [142]. Furthermore, EVs are being explored as therapeutic vectors, leveraging their innate ability to cross the BBB, deliver nucleic acids, proteins, or drugs and modulate target cells with reduced immunogenicity compared to synthetic nanoparticles [141, 143].

Collectively, the current evidence positions EVs as both key players in neurodegeneration and innovative tools for diagnostics and therapy, bridging fundamental research and clinical applications in neurology. Ongoing studies are focused on defining cargo selection mechanisms, optimizing EV engineering for targeted delivery and validating EV‐based biomarkers in longitudinal clinical studies.

EVs are key mediators of intercellular communication also in cancer and contribute extensively to tumor progression, metastasis, immune evasion, therapy resistance, and biomarker potential. Tumor‐derived EVs carry a rich cargo of proteins, nucleic acids (including miRNAs and oncogenic transcripts), lipids, and metabolites that modulate recipient cells and reshape the tumor microenvironment to support malignant behavior; this content confers mesenchymal properties to epithelial cells and promotes the initial phase of tumor metastasis, when in situ tumor cells acquire the ability to migrate out of the primary tumor site, invading basement membrane, and enter the circulatory system [144, 145], ensuring communication between the tumor and its microenvironment.

Moreover, they promote ECM degradation, angiogenesis, and the formation of premetastatic niches that facilitate distant organ colonization by circulating tumor cells (CTCs) [146]. EVs also alter cellular metabolism in cancer cells and host tissues, contributing to the metabolic reprogramming characteristic of malignancy and supporting growth under hostile conditions [147].

In colorectal and other solid tumors, EVs influence immune suppression by recruiting regulatory immune cells and fostering an immunosuppressive microenvironment, while transferring cargo that can drive therapy resistance and enhance survival pathways in recipient cells [148]. Clinically, EVs released into body fluids emerge as powerful liquid biopsy biomarkers because their stable molecular contents reflect tumor subtypes and molecular characteristics more accurately than traditional methods, enabling early detection and monitoring of disease progression and response to therapy [149, 150, 151].

Collectively, recent studies highlight the vital role of EVs in various aspects of cancer biology, emphasizing their functional contributions to malignancy and their potential as diagnostic and therapeutic tools in precision oncology.

## 5 Mechanisms of Selective Cargo Loading: A Focus on miRNAs

As previously introduced, EVs contain various molecules, including proteins, lipids, DNA, coding, and noncoding RNAs. Concerning noncoding RNAs, miRNAs have attracted considerable attention not only for their functional role in post‐transcriptional regulation of gene expression but also because, among small RNAs, miRNAs are more enriched in EVs than in their parental cells [152].

The presence of specific miRNAs in EVs, which does not necessarily mirror their expression in the EV‐producing cells, indicates the existence of selective miRNA‐loading mechanisms into EVs [153, 154]. Despite the limited knowledge of these molecular events, current scientific evidence has provided new insights into this biological phenomenon, including the role of specific proteins (Tables 3 and 4).

**TABLE 3:** List of proteins involved in miRNA loading into EVs in the specified cell types.

Protein	Cell type	Role	References
nSMase2	Cancer and mesenchymal stem cells Metastatic breast cancer cells	miRNA loading into EVs	Kosaka et al., 2013 [155] Singh et al., 2014 [156] Zhu et al., 2018 [157]
Vps4A	HCC cells Kidney cells	miRNA loading into EVs	Wei et al., 2015 [158] Jackson et al., 2017 [159]
KRAS	Colorectal cancer cells	miRNA loading into EVs	Cha et al., 2015 [160]
Substance P/NK1R receptor	Colon cells	miRNA loading into EVs	Bakirtzi et al., 2019 [161]

**TABLE 4:** List of RBPs involved in miRNA loading into EVs or their intracellular retention in the specified cell types.

RBP	Cell type	Role	References
SYNCRIP	Hepatocytes	miRNA loading into EVs	Santangelo et al., 2016 [7]
PCBP2	Hepatocytes	miRNA cell retention	Marocco et al., 2025 [6]
YBX‐1	Endothelial progenitor cells Kidney cells	miRNA loading into EVs	Lin et al., 2019 [162] Shurtleff et al., 2016 [163]
MEX3C	Kidney cells	miRNA loading into EVs	Lu et al., 2017 [164]
MVP	Colon cancer cells	miRNA loading into EVs	Teng et al., 2017 [165]
La	Breast cancer cells	miRNA loading into EVs	Temoche‐Diaz et al., 2019 [166]
IGF2BP1	Melanoma	miRNA loading into EVs	Ghoshal et al., 2019 [167]
hnRNP A2B1	T‐lymphocytes	miRNA loading into EVs	Villarroya‐Beltri et al., 2013 [8]
ALYREF	Hepatocytes Brown adipocytes	miRNA loading into EVs	Garcia‐Martin et al., 2022 [5]
FUS	Hepatocytes Brown adipocytes	miRNA loading into EVs	Garcia‐Martin et al., 2022 [5]

Abbreviations: ALYREF, Aly/REF export factor; FUS, fused in sarcoma; hnRNP A2B1, heterogeneous nuclear ribonucleoprotein A2/B1; IGF2BP1, insulin‐like growth factor 2 mRNA‐binding protein 1; MEX3C, mex‐3 RNA‐binding family member C; MVP, major vault protein; YBX‐1, Y‐box binding protein 1.

Neutral sphingomyelinase (nSMase2) was the first molecule recognized for its role in EV miRNA sorting; it regulates the EV‐export of miR‐210 in cancer and MSCs, as well as miR‐10b in metastatic breast cancer cells [156, 157]. Additionally, inhibiting nSMase2 decreases the EV‐export of miR‐16 and miR‐146a, while its overexpression increases their EV loading without affecting their intracellular levels [155].

The vacuolar protein sorting‐associated protein 4 (Vps4A) is typically described as a regulator of endosomal trafficking, but recent findings suggest that it also contributes to the EV‐export of miR‐27b‐3p and miR‐92a‐3p in hepatocellular carcinoma (HCC) [158]. Furthermore, its inhibition in HEK293 cells reduces the loading of miR‐92a and miR‐150 [159].

The oncogene KRAS (in both wild‐type and mutated forms) can also play a role in this process: miR‐100 shows higher expression in mutant KRAS‐derived exosomes, miR‐10b is significantly higher in wild‐type KRAS‐derived exosomes, while miR‐320 families are expressed in both wild‐type and mutant‐derived exosomes [160]. Substance P (SP) and its receptor, NK1R, are involved in the expression of several miRNAs in the cytosol of colon cells. Recent findings indicate that SP/NK1R signaling influences the regulation of miRNA sorting into EVs; notably, in colon cells, exosomal miR‐21 levels are higher in response to SP signaling than in unstimulated cells [161]. Additionally, certain RNA sequence modifications also affect this process. In human B cell‐derived exosomes, the distribution of miRNAs depends on post‐transcriptional modifications at the 3′ end. Specifically, 3′ end adenylation promotes miRNAs’ cellular retention, while 3′ end uridylation promotes miRNAs’ export [168].

Here, some RBPs, RNA motif sequences and RNA epitranscriptomic modifications are considered in detail as key determinants for the selectivity of miRNA export/retention dynamics of miRNAs into EVs.

### 5.1 RNA‐Binding Proteins as Key Mediators (SYNCRIP, PCBP2, and Beyond)

Since the initial discovery of RNA export into EVs, RBPs have emerged as central players [169]. In particular, the heterogeneous nuclear ribonucleoproteins (hnRNPs) represent an evolutionarily conserved family of ubiquitously expressed RBPs that share several nucleic acid targets and molecular functions.

**FIGURE 3:**
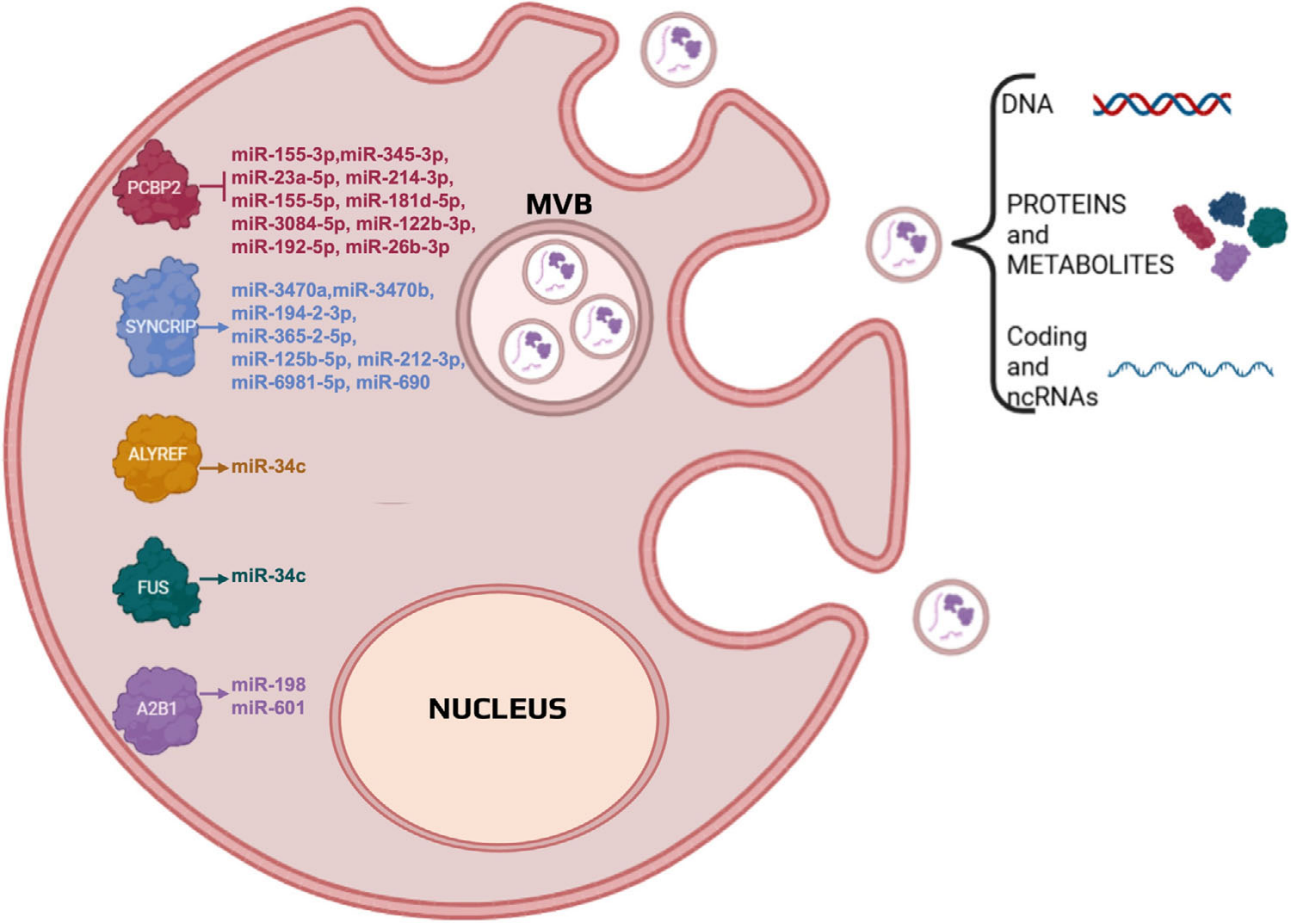
Schematic illustration of EV release highlighting EV cargo and RNA‐binding proteins involved in miRNA intracellular retention or small EV‐export. RBPs are shown on the left: arrows indicate the RBP‐mediated export of the reported miRNAs, and T‐bars represent the RBP‐mediated inhibition of EV‐export for the reported miRNAs. MVB, multivesicular body. Small circles depict EVs enclosing macromolecules as defined on the right.

As further regulation of their roles, hnRNPs can undergo post‐translational modifications such as methylation, phosphorylation, ubiquitination, and sumoylation [170], which influence their localization and activity. Among the various RBPs identified in the scientific literature, many are recognized to play a key role in regulating miRNA loading into EVs (Table 4 and Figure 3).

SYNCRIP and PCBP2, besides their pleiotropic roles across distinct cell types, represent key players in miRNA‐EV delivery.

SYNCRIP, also known as hnRNPQ, is an hnRNP conserved across eukaryotes and expressed in different splicing isoforms, with the most common being hnRNP Q3, hnRNP Q2, and hnRNP Q1.

It plays distinct roles in regulating gene expression at both post‐transcriptional and translational levels; specifically, it is involved in RNA splicing, editing and cytoplasmic transport. It also participates in the degradation and regulation of internal ribosome entry site (IRES)‐dependent translation [171, 172, 173]. Due to its pleiotropic functions, SYNCRIP affects several biological processes such as neural and muscular development and the homeostasis of hematopoietic stem and progenitor cells [174, 175].

In HCC, SYNCRIP is considered an important prognostic marker since high levels of SYNCRIP are associated with a poor prognosis (source: The Human Protein Atlas, proteinatlas.org). Furthermore, the role of SYNCRIP as a regulator of EMT/MET (epithelial‐to‐mesenchymal transition/mesenchymal‐to‐epithelial transition) dynamics has recently been uncovered in liver cells, where the involvement of transcription factors and ncRNAs has been previously described [144, 145, 176, 177, 178, 179]. Specifically, SYNCRIP acts as a “mesenchymal” gene in hepatocytes, being induced by TGFβ and positively regulating EMT. Its silencing limits the induction of mesenchymal gene expression and the mesenchymal program, while maintaining the epithelial and hepatic phenotypes at the molecular and morphological levels. Furthermore, in HCC invasive cells, SYNCRIP knockdown induces a MET at both molecular and phenotypic levels, negatively regulating mesenchymal gene expression, inducing epithelial markers and significantly impairing cell migration. Moreover, during EMT/MET dynamics, SYNCRIP knockdown alters the expression of a set of pro‐ or anti‐EMT miRNAs, suggesting a potential role for this protein in regulating their transcription [180].

**FIGURE 4:**
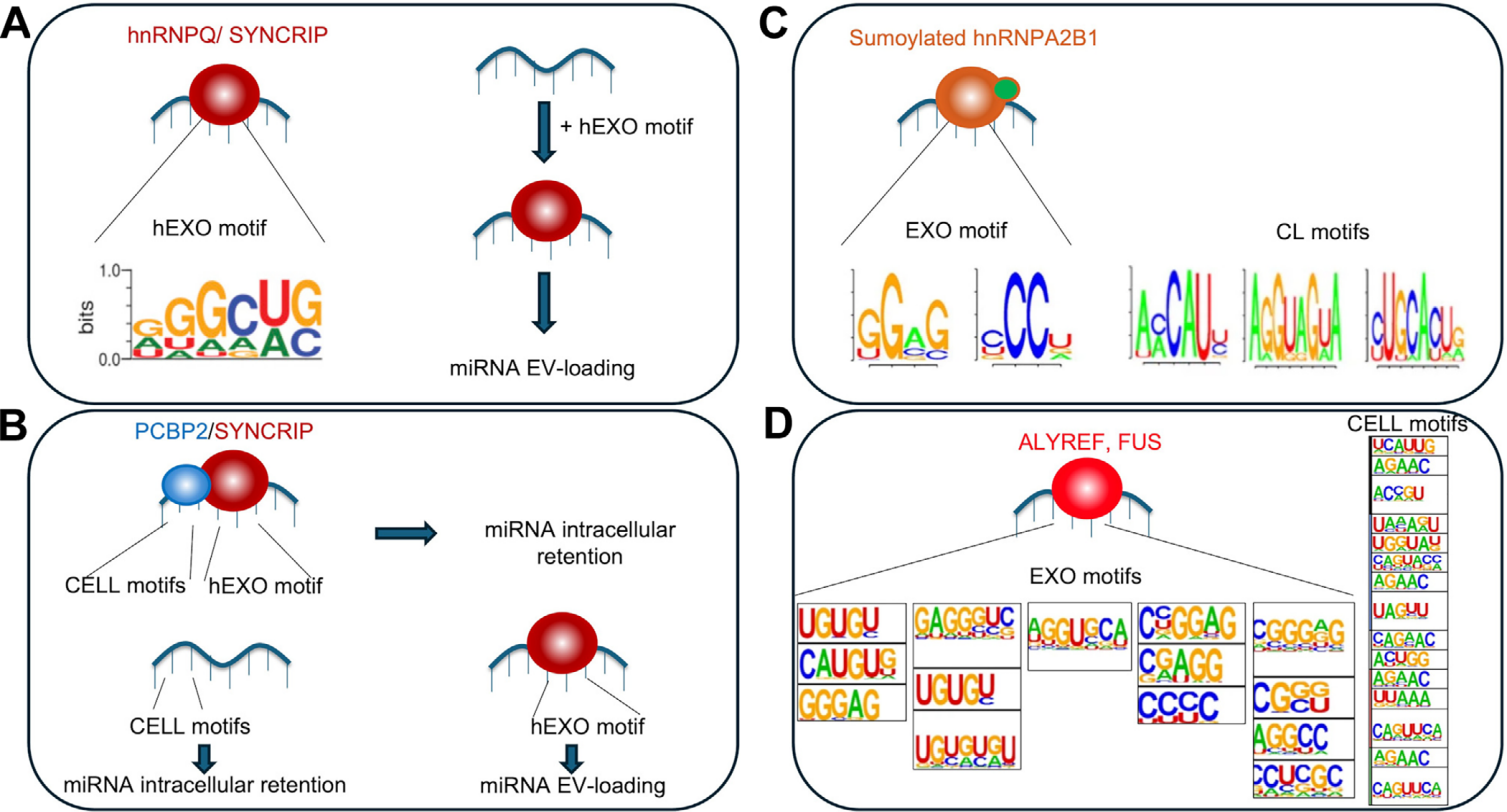
Schematic representation of the molecular mechanisms mediated by RNA‐binding proteins and motif sequences involved in miRNA intracellular retention or EV‐export. Consensus motifs for the indicated proteins are reported as in Santangelo [7] (A), Marocco [6] (B), Villarroya‐Beltri [8] (C), and Garcia‐Martin [5] (D). RNA‐binding proteins are depicted as circles and shown at the top of each model in the corresponding color. Motif sequences recognized by each RBP are presented as logos according to [5, 6, 7, 8].

Interestingly, the interaction between the SYNCRIP protein and target RNAs is mediated by the three RRMs as well as by the N‐terminal region, known as the N‐terminal unit for RNA recognition (NURR). This domain interacts with the miRNA hEXO motif, a specific consensus sequence found in EV‐loaded miRNAs in the extra‐seed position [7] (Figure 4A) and is responsible for SYNCRIP‐miRNA recognition [181]. Both the NURR and RRM domains act together to mediate the interaction between SYNCRIP and target RNAs [181].

Poly(rC)‐binding protein 2 (PCBP2), also known as hnRNPE2, is an RBP that recognizes and interacts with poly(C) sequences in a sequence‐specific manner. PCBP2 also has a post‐transcriptional regulatory role due to its ability to interact with pyrimidine‐rich regions, as in the case of the α‐globin mRNA UTR, enhancing mRNA stability. Moreover, it has the ability to modulate gene expression at a translational level; in fact, it can enhance the translation of c‐Myc by binding to the c‐Myc mRNA IRES both in vivo and in vitro [182]. Notably, PCBP2 is also a key player in various human cancers. Its expression correlates with poor prognosis, high rates of cell proliferation and resistance to conventional chemotherapy in HCC [183]. Mechanistically, PCBP2 interacts with the Yes‐associated protein (YAP), cooperating in tumor growth [184].

In glioblastoma, it is highly expressed and associated with poor prognosis, clinical stages and histological grade [185]; moreover, it can influence tumor cell proliferation, migration, invasion, EMT and apoptosis [185, 186, 187].

More recently, PCBP2 has been shown to be involved in retaining specific miRNAs within cells [6]. As previously described, several motif sequences have been identified as determinants for miRNA export in EVs or for their intracellular retention. In miRNA EV‐sorting, the characterization of consensus sequences was enhanced by identifying RBPs that specifically recognize these motifs. However, the proteins bound to cell retention motifs remain uncharacterized. In a recent study, PCBP2 was found to act as a miRNA retainer by recognizing and binding to cell‐retention motifs (Table 4). More interestingly, its function requires interaction with the EV‐loader SYNCRIP, which is bound to the hEXO motif. Consequently, PCBP2 acts as a dominant negative regulator of SYNCRIP, impairing SYNCRIP‐dependent miRNA EV‐sorting (Figure 4B).

Additional RBPs implicated in miRNA EV‐export are described below.

Y‐box binding protein 1 (YBX‐1) is a multifunctional RNA/DNA‐binding protein from the Y‐box transcription factor family involved in transcription, RNA splicing and transport, translation and DNA repair. Experimental findings report that it is overexpressed in many cancers and associated with tumor progression and poor prognosis. Moreover, it also plays key roles in embryonic development and tissue differentiation [188]. Notably, it is also involved in packaging miR‐133 into exosomes after hyperoxia/reperfusion. YBX‐1 silencing decreases miR‐133 export into exosomes without affecting its expression in the cytoplasm, while YBX‐1 overexpression triggers miR‐133 loading into exosomes [162]. Moreover, YBX‐1 is also required for exporting miR‐223 in HEK293T‐derived exosomes, a process that does not depend on sequence recognition but solely on the interaction between this miRNA and the YBX‐1 internal cold shock domain [163].

MEX3C functions as an RNA‐binding E3 ubiquitin ligase essential for RNA degradation; it acts as a post‐transcriptional repressor, is deregulated in various cancers, influences tumor‐related processes such as apoptosis and immune evasion, and is emerging as a potential prognostic marker and target for cancer therapy [189]. However, there is evidence that it also acts as a miRNA loader into EVs, as shown by its silencing affecting the export of miR‐451a [164] in kidney cells.

Major vault protein (MVP) is a ribonucleoprotein that modulates the tumor‐associated macrophage M2 transcriptional program [190]. It is required for the transport of RNAs from the nucleus to the cytoplasm and has been shown to be involved in EV miRNA sorting. Silencing MVP in CT26 colon cancer cells negatively impacts the loading of miR‐193a into exosomes. The selectivity of this mechanism is underscored by the fact that this phenomenon is not observed with other miRNAs, such as miR‐126a [165].

The La protein is another RBP that functions as a transcription factor for the RNA polymerase III, shuttling between the nucleus and the cytoplasm. It is often overexpressed in various cancers, where it promotes tumor progression, cell survival, and resistance to chemotherapy by enhancing the processing and translation of tumor‐promoting and antiapoptotic factors, making it a potential therapeutic target [191]. Cytosolic La‐depleted cells are characterized by a significant reduction in miR‐122 packaging into EVs produced by breast cancer cells [166].

IGF2BP1 is an oncofetal RBP implicated in melanoma progression and metastasis. In vivo studies showed that its depletion markedly reduced lung metastasis through effects mediated by tumor‐derived EVs. While IGF2BP1 did not alter EV production or uptake, omics analyses revealed significant changes in EV‐RNA cargo, including miRNAs, indicating a role in selective loading. These findings support IGF2BP1 as a regulator of miRNA incorporation into EVs that influences their prometastatic activity [167].

The hnRNP A2B1 is a multifunctional RBP involved in RNA metabolism, gene expression, and translation. Notably, its dysregulation can lead to inflammation, immune responses and cancer progression; these factors make it an emerging molecular target for therapeutic intervention [192]. Notably, the sumoylated form of hnRNPA2/B1 was discovered to recognize short RNA motifs (EXO motifs) along miRNAs and to vehicle them into exosomes released by T‐lymphocytes [8] (Figure 4C). Moreover, hnRNPA2/B1 also participates in packaging circEHD2, leading to an EV‐circEHD2‐mediated transformation of fibroblasts into cancer‐associated fibroblasts (CAFs) [193] and in packaging circCCAR1, which causes dysfunction of CD8+ T cells [194].

AlyRef (Aly/REF export factor) is an RBP that regulates mRNA processing, stability and nuclear‐cytoplasmic transport, promoting cancer cell proliferation, metastasis, and drug resistance. Moreover, it is emerging as a potential prognostic biomarker and therapeutic target despite limited clinical validation [195]. Mechanistically, AlyRef can recognize G‐quadruplex RNAs, and the presence of G‐rich EXO motifs on several miRNAs allows it to recognize and to export specific miRNAs into the EVs [5, 196] (Figure 4D).

Fused in sarcoma (FUS) protein is another RBP that normally regulates RNA processing, splicing and DNA repair; moreover, it contributes to cancer progression through pro‐oncogenic signaling and altered transcriptional control [197], influences cardiovascular pathology by modulating the alternative splicing of proliferation‐related genes in atherosclerosis [198] and drives neurodegeneration [199]. It is also involved in miRNA sorting into EVs through the recognition of EXO motifs [5] and also facilitates the loading of circRNAs into EVs in neurons under hypoxia [200] (Figure 4D).

### 5.2 RNA Motifs and Cis‐Regulatory Elements

Most RBPs involved in miRNA export/retention exert their functions by recognizing specific RNA motif sequences (Table 5).

**TABLE 5:** List of RNA consensus motifs bound by the indicated RBPs, exerting either miRNA EV‐export or intracellular retention functions in the specified cell types.

Sequence motif	Cell type	Recognizing RBP	Role	References
UAGGG(A/U) AGAGGG	Cancer‐associated fibroblast Leukemia cells	hnRNP A1	miRNAs EV‐export	Qin et al., 2019 [201] Zhang et al., 2020 [202] Gao et al., 2019 [203]
AGAGGG	Pancreatic cancer cells	SRSF1	miRNAs EV‐export	Xu et al., 2020 [204]
GGAG	T‐lymphocytes	hnRNP A2B1	miRNAs EV‐export	Villarroya‐Beltri et al., 2013 [8]
GGCU	Hepatocytes	SYNCRIP	miRNAs EV‐export	Santangelo et al., 2016 [7]
CGGGAG	Brown adipocytes, hepatocytes	ALYREF/FUS	miRNAs EV‐export	Garcia‐Martin et al., 2022 [5]
AUU(A/G)	Hepatocytes	PCBP2	miRNAs intracellular retention	Marocco et al., 2025 [6]

Abbreviations: ALYREF, Aly/REF export factor; FUS, fused in sarcoma; hnRNP A1, heterogeneous nuclear ribonucleoprotein A1; hnRNP A2B1, heterogeneous nuclear ribonucleoprotein A2/B1; RBP, RNA‐binding protein; SRSF1, serine/arginine‐rich splicing factor 1.

The protein hnRNPA1 recognizes the RNA motif UAGGG(A/U), promoting the export of miR‐196a into EVs derived from CAFs [201] and triggering the packaging of miR‐522 upon deubiquitylation by USP7 [202]. Moreover, hnRNPA1 promotes the loading of miR‐320 through recognition of the AGAGGG motif in leukemia cells [203].

The SRSF1 protein can bind to a 6 bp shared motif in exosomal miRNAs and regulates miRNA loading in EVs produced by pancreatic cancer cells [204].

In primary T cells, two RNA motifs highly represented in exosomal miRNAs (EXO motifs) and three motifs highly represented in miRNAs retained within the cells (CL motifs) have been identified. These motifs determine the fate and localization of miRNAs, whether inside the cells or into the EVs (Figure 4C). Interestingly, inserting an EXO motif into the cell‐retained miR‐17 causes its export into EVs, while adding a CL motif to the EV‐loaded miR‐601 results in its cellular retention [8].

In addition, hnRNPA2B1 specifically recognizes the RNA motif AGG/UAG and binds to the MVs‐associated miR‐17 and miR‐93, both of which share this consensus sequence [205].

Moreover, a cellular and EV‐packaged miRNA repertoire has been characterized in hepatocytes, expanding the characterization of further proteins involved in miRNA sorting into EVs; specifically, a profile of EV‐enriched miRNAs was identified, revealing that the RBP SYNCRIP specifically interacts with several EV‐enriched miRNAs sharing the consensus sequence GGCU, known as the hEXO motif and is involved in packaging bound miRNAs into EVs (Figure 4A). In fact, inserting the hEXO sequence into cell‐retained miR‐29b enables direct interaction with SYNCRIP and thus facilitates its sorting into EVs [7].

To summarize, SYNCRIP and hnRNPA2B1 can recognize different miRNAs in a sequence‐specific manner and export them. hnRNPA2B1 can recognize and interact with miRNAs that contain both EXO and hEXO motifs, but not miRNAs that contain only the hEXO sequence (e.g., miR‐3470). Similarly, SYNCRIP does not bind to miRNAs embedding only the GGAG EXO motif.

Furthermore, hnRNPA2B1 silencing does not negatively impact the export in EVs of GGCU containing miR‐3470a and miR‐194‐2‐3p, while the number of EVs‐miRNAs embedding the GGAG sequence is significantly reduced [7, 181].

Then, it has been confirmed that the miRNA populations loaded in small EVs (sEVs) differ significantly from those expressed by the cells of origin. Moreover, analysis of miRNA profiles across five different cell lines revealed that different miRNAs are enclosed in sEVs depending on the producing cell line. Furthermore, through in silico analysis, between one and four 4–7‐nucleotide‐long sequence motifs have been identified for each cell line that are enriched in sEV miRNAs and are named EXO motifs. Additionally, two to five 4–5‐nucleotide RNA motifs found in miRNAs retained within cells have been identified and called CELL motifs [5] (Figure 4D).

The identified motifs, which govern miRNA distribution between the intracellular compartment and EVs, are both necessary and sufficient to direct their localization. Indeed, the introduction of the CELL motif AGAAC into the sequence of miR‐431‐5p (a previously defined sEV‐enriched miRNA) caused a decrease in its loading into EVs, while mutating the same motif in another miRNA, miR‐140‐3p, increased its sEV‐export. As a mirror approach, the EXO motif was also inserted into cell‐retained miRNAs, miR‐34c‐5p and miR‐26a; in both cases, including this motif enhances their sEV loading. Notably, the same group that designed these chimeric miRNAs identified (through a mass spectrometry‐based approach) two additional proteins, ALYREF and FUS. Both proteins interact with miR‐34c and miR‐26a when the CGGGAG EXO motif is present in the miRNA sequence. Functionally, silencing ALYREF and FUS counteracts miRNA enrichment in sEVs, demonstrating their crucial role in miRNAs’ EV‐export. Once loaded into EVs, miRNA produced by donor cells can act on their molecular targets in recipient cells by negatively regulating mRNA stability or translation [5].

### 5.3 RNA Modifications in EV Loading

RNA modifications are chemical modifications that occur on different classes of RNA molecules, including mRNAs, as well as rRNAs, tRNAs, lncRNAs, miRNAs, piRNAs, and circRNAs. These chemical modifications include 2′‐O‐methylation, pseudouridine (Ψ), N6‐methyladenosine (m6A), 5‐methylcytosine (m5C), 5‐hydroxymethylcytosine (hm5C), N1‐methyladenosine (m1A), N6, 2′‐O‐dimethyladenosine (m6Am), 2′‐O‐methylation (Nm), and N7‐methylguanosine (m7G). RNA modifications are dynamically regulated since they can be added by enzymes known as writers and removed by the so‐called erasers [206].

m6A modification is the most extensively studied and occurs on specific RNA motifs (RRACH motifs, where R = A/G and H = A/C/U), initially detected in the 3′ UTRs, 5′ UTRs, and in the coding sequences of mRNAs [207], or in DRACH motifs (D = A/G/U, R = A/G, H = A/C/U), or RAC motifs (R = A/G, H = A/C/U) [208]. m6A is added by the writer complex, which includes methyltransferases METTL3/14 and cofactor proteins such as Wilms tumor 1‐associated protein (WTAP) [206].

Furthermore, a group of RBPs, known as readers, recognizes and binds specific modified RNAs and exerts specific molecular and biological functions. These RBPs, as m6A readers, play various roles in RNA metabolism, affecting splicing, RNA processing, RNA stability/degradation and translational regulation [206].

**FIGURE 5:**
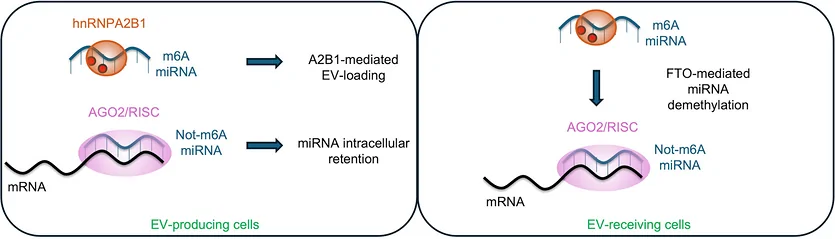
Schematic illustration of the molecular mechanism connecting miRNA methylation status with intracellular activity or EV‐export. Left: Depiction of miRNA fate based on its methylation status during the EV production. Top: Methylated miRNAs bind to RBP A2B1 and are loaded into EVs. Bottom: Not methylated miRNAs interact with the RISC complex and exert their role in the intracellular compartment as post‐transcriptional regulators of gene expression. Right: Illustration of the demethylation step necessary for EV‐delivered miRNAs to function in recipient cells. FTO‐mediated miRNA demethylation is required to remove methylation from EV‐carried miRNAs, allowing their interaction with the RISC complex and their function in recipient cells.

This epitranscriptomic modification also plays a role in the biogenesis of miRNAs; it has been reported that miRNA processing is affected by m6A modification, that NSUN2 (NOP2/Sun RNA methyltransferase 2) negatively affects miR‐125b maturation, and that m6A is crucial for the recognition of primary miRNAs (pri‐miRNAs) [209]. Additionally, pri‐miRNAs are recognized by hnRNPA2B1 for recruitment and interaction with DiGeorge syndrome critical region 8 (DGCR8) for processing [210]. In another work, it was initially suggested that m6A modifications could influence not only miRNA biogenesis but also their export into EVs [211]. A recent work has provided insight into the epitranscriptomic mechanism regulating miRNA export into EVs [9]. In this study, it has been reported that m6A modification is selectively accumulated along mature miRNAs. Notably, this epitranscriptomic modification impairs AGO2 binding and, in turn, the functional activity of miRNAs within the cell, while it enhances their binding to the m6A reader and EV loader hnRNPA2B1. To summarize, m6A inhibits miRNAs’ function inside the cell and promotes their loading into EVs, thus affecting their fate. Moreover, EV recipient cells can utilize EV miRNA content through FTO‐dependent demethylation, thereby restoring miRNA activity in EV‐target cells (Figure 5). Interestingly, analysis of the human and murine miRNome revealed that many miRNAs share a high percentage of m6A sites, indicating they are methylable in principle. This suggests that the methylation status can be precisely regulated depending on cell type, tissue and the cell's pathophysiological condition. Finally, in a recent paper, 22 RNA modifications were identified on EV‐RNAs, many of which are downregulated in tumor‐derived EVs and linked to cancer recurrence [212]. Consistently, the downregulation of the m6A demethylase ALKBH5 in this cellular system leads to increased m6A levels, which in turn alter the effects of colon cancer‐derived EVs, particularly regarding the regulation of the immune response.

**FIGURE 6:**
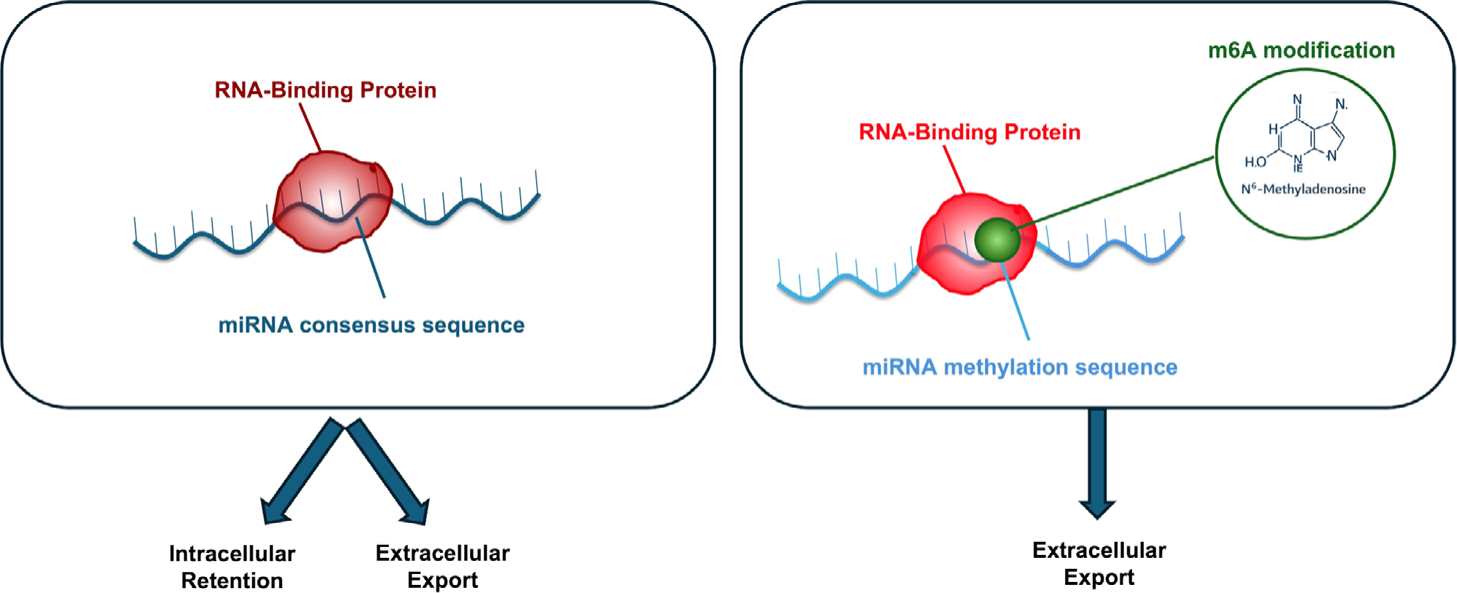
Integrated model of miRNA EV‐cargo loading considering the crosstalk among RBPs, miRNA sequence determinants and RNA modifications. Left: Depiction of RBP directly binding to miRNAs’ consensus sequences, determining miRNA fate (intracellular retention vs. EV‐export). Right: Depiction of the role of miRNA methylation, required for the binding of specific RBPs involved in miRNA EV‐export. m6A modification is shown as a green circle on the miRNA sequence (in blue).

Overall, this evidence highlights the complexity of miRNA EV‐cargo loading, which involves an integrated mechanism that includes RBPs, sequence motifs acting as a barcode directly recognized by the RBPs and epitranscriptomic modifications that further enhance RBP binding to miRNAs embedding these motif sequences (Figure 6).

### 5.4 Computational Prediction of Sorting Determinants

Recent advancements in computational biology, especially in deep learning, have significantly expanded the tools available for RNA motif discovery and RNA–protein interaction (RPI) prediction, offering valuable opportunities for studying EV‐cargo sorting. A wide range of motif‐mining frameworks has been developed to identify both sequence‐based and structure‐aware RNA motifs, including approaches that incorporate network‐based and multistructure motifs. These methodologies facilitate the detection of conserved cis‐regulatory elements that may drive selective RNA packaging within EVs, even in the presence of structural diversity [213, 214]. In parallel, deep learning‐based models for RNA–protein binding prediction have achieved near‐base‐resolution accuracy by combining sequence features, secondary structure information, thermodynamic context and more recently, language model embeddings. Tools such as transformer‐based architectures and hybrid convolutional‐recurrent networks enable protein‐specific, transcript‐wide prediction of RNA‐binding sites across multiple RBPs, even in cross‐protocol and cross‐batch settings. Notably, several platforms focus on interpretability and network‐guided learning, helping identify RBPs that may be involved in the selective EV loading of RNAs [215, 216, 217].

Beyond binary interaction prediction, emerging AI‐driven frameworks integrate multiomic and network‐level data to model RPI landscapes on a large scale. These methods, combined with advances in RNA‐ligand and small‐molecule interaction predictions, suggest that AI‐based approaches could be extended to model competitive or cooperative binding events that affect RNA function and subcellular localization. Despite these developments, the use of these computational tools in EV biology remains limited. Systematic integration of motif discovery, RPI prediction, and EV transcriptomic datasets could provide a powerful, hypothesis‐generating framework to uncover sorting determinants and guide experimental validation in future studies [218, 219, 220].

## 6 Therapeutic Opportunities

### 6.1 Native EVs as Therapeutic Agents (e.g., MSC‐EVs in Regenerative Medicine)

Several pieces of evidence support the concept of EVs as precision therapeutic vectors for delivering small molecules, proteins, and nucleic acids, including therapeutic RNAs. Compared to synthetic nanoparticles and viral vectors, EVs offer advantages such as improved biological compatibility, the ability to cross biological barriers and reduced immunogenicity. Advances in EV surface engineering, cargo loading techniques and bioinspired synthetic vesicles are expanding their applicability in targeted drug delivery and gene therapy, positioning EVs as a promising next‐generation delivery system [221, 222, 223, 224, 225]. Understanding the molecular mechanisms and the molecular actors involved in specific RNA cargo loading into EVs is essential for manipulating these processes. This knowledge is crucial for producing a population of EVs with targeted RNA cargo tailored for therapeutic purposes. Currently, it is recognized that EVs do not export cargo randomly.

In recent times, great attention has been focused on MSCs that are safe, easy to manipulate in the laboratory, have a high amplification potential and possess significant immunosuppressive properties. It has been reported that they can be used to treat autoimmune diseases such as graft‐versus‐host disease (GVHD), a severe complication of allogeneic transplantation in which transplanted donor immune cells attack the recipient's tissues.

Notably, the powerful immunomodulatory effects of these cells are largely due to the RNA content of MSC‐derived EVs. In fact, MSC‐derived exosomes are abundant in miRNAs and mRNAs that exhibit anti‐inflammatory, antiapoptotic, antioxidant, immunoregulatory, and proangiogenic functions, promoting tissue repair across various tissues and organs [226, 227], making them a valuable tool for therapeutic interventions.

Regarding GVHD, one interesting approach is the use of MSC‐derived exosomes to ameliorate symptoms [228]. Although the main therapy involved MSC injections in patients, utilizing MSC‐derived exosomes as an alternative appears to offer the same benefits and could be a promising substitute for MSC transplantation.

### 6.2 Engineered EVs for Drug Delivery: Loading Strategies and Surface Engineering

For the treatment of neurological diseases, several studies report the effectiveness of the use of EVs in therapeutic applications. EVs enriched with miR‐133b have been shown to promote neurovascular recovery in neurons and astrocytes [229], EVs enriched with miR‐219 stimulate oligodendrocyte remyelination [230], while EVs enriched with miR‐125b induce MSCs to differentiate into neurons [231] or enhance Aβ peptide clearance in AD [232].

To further enhance these therapeutic effects, EVs can be engineered through specific loading strategies, including passive incubation, chemical transfection, electroporation, or endogenous loading via overexpression of therapeutic RNAs in donor cells [233]. These approaches allow precise control over the RNA cargo and improve the efficiency of delivery to target neural populations.

For example, engineered exosomes designed to specifically deliver siRNAs for BACE1 (β‐secretase) or γ‐secretase represent effective strategies for reducing the levels of these enzymes involved in Aβ‐peptide production [234]. Additionally, a key advantage of therapeutic EVs in treating neurological disorders is their ability to cross the BBB, a feat much harder to achieve with other drug delivery strategies [235]. In vivo studies indicate that delivering BACE1 siRNA via exosomes is effective for treating AD in mice [94].

Beyond cargo loading, surface engineering (such as the display of targeting peptides, antibodies, or ligand–receptor pairs) can further increase BBB penetration and enhance the selective uptake of EVs by neurons, microglia, or oligodendrocytes [236]. Strategies such as Lamp2b‐fusion peptides, RVG peptides, or integrin‐based targeting have been widely explored to improve brain tropism and therapeutic precision (Figure 2, right panel).

Another benefit of using EVs is their role as delivery systems for RNA drugs. In this case, EVs can be purified from donor cell media in the laboratory and then transfected or electroporated with therapeutic RNA molecules (such as siRNAs, mimic miRNAs, antagomiRs, etc.) [237]. It is also possible to load EV‐producing cells with therapeutic RNAs or to transfect them with a plasmid encoding for the therapeutic RNAs, which are then naturally sorted into EVs by endogenous systems. Once produced, EVs can be easily purified for therapeutic use.

For example, from a single patient, blood sampling can be used to culture cells ex vivo, inducing their differentiation into mature macrophages or DCs. These cells can then be engineered with therapeutic RNAs or transfected with a plasmid encoding the desired RNA molecule to be packed into EVs. After purifying these EVs and performing proper quality control, they can be reintroduced into the same patient to create a personalized EV‐RNA therapy [237] (Figure 2, right panel).

This autologous approach, combined with customized loading strategies and surface engineering, represents one of the most promising avenues for next‐generation precision nanomedicine.

Importantly, engineered EVs can also be functionalized on their membrane surface (Figure 2, right panel) to improve circulation time, reduce clearance by macrophages, or enhance homing to specific tissues. Approaches include PEGylation, insertion of synthetic lipid‐anchored ligands, or genetic engineering of donor cells to express membrane‐tethered targeting motifs [238].

### 6.3 Therapeutic Applications: Immunotherapy, Oncology, and Tissue Repair

EVs can be used to deliver RNAs as therapeutic molecules to induce an immunomodulatory response (e.g., in the case of vaccines [239, 240]) or for immunosuppression, tissue regeneration [241, 242], and molecular diagnosis [243]. Their ability to encapsulate and protect RNA molecules from degradation, combined with their natural tropism toward specific cell types, makes EVs highly versatile therapeutic tools across multiple disease contexts [244] (Figure 2, right panel).

In immunotherapy, EVs contribute to both immune activation and immune suppression. It has been reported that exosomes derived from IFNγ‐treated cells contain anti‐inflammatory RNAs with immunomodulatory effects in multiple sclerosis (MS), as shown through RNA sequencing [245]. These findings highlight the capacity of EVs to modulate immune pathways in immune diseases. Additionally, EV‐based RNA delivery is being explored to enhance antigen presentation and improve vaccine efficacy [246], leveraging their intrinsic biocompatibility and ability to interact with immune cells.

In the same field of EV‐based therapeutic strategies, it has been reported that exosome‐miRNAs derived from MSCs have significant therapeutic benefits against spinal cord injury (SCI), modulating oxidative stress, apoptosis, and inflammation [247].

These regenerative effects extend to other tissues, as MSC‐EVs promote angiogenesis, reduce fibrosis, and support cellular survival in damaged environments [248].

Moreover, engineering MSC‐derived exosomes containing specific miRNAs offers a promising approach to regulate target gene expression and provide a therapeutic effect [249].

In oncology, EVs are being investigated as carriers of therapeutic RNAs capable of reprogramming tumor cells or modulating the tumor microenvironment [250]. Their natural ability to transfer functional miRNAs and siRNAs enables the suppression of oncogenic pathways, while engineered EVs can be tailored to deliver tumor‐suppressive RNAs or to silence genes involved in proliferation, invasion, or drug resistance [251].

EVs also hold promise as vehicles for cancer immunotherapy, as they can deliver immunostimulatory RNAs to DCs or T cells, thereby enhancing antitumor immune responses [252].

Overall, the ability of EVs to modulate immune responses, influence tumor biology, and promote tissue repair underscores their broad therapeutic potential and supports their development as next‐generation RNA delivery systems.

### 6.4 Emerging EV‐Mimetics and Synthetic Alternatives

Alongside efforts to improve the production, standardization, and engineering of natural EVs, a new class of platforms (collectively referred to as EV‐mimetics [253] or synthetic EV [254] alternatives) has rapidly emerged.

These systems include biomimetic nanoparticles [255, 256], engineered polymers [257], advanced liposomes [258], and cell membrane‐derived nanovesicles [259], all designed to replicate the structural features, biodistribution patterns, and delivery capabilities of natural EVs.

Their development aims to overcome intrinsic limitations of biological EVs by providing carriers that are more homogeneous, scalable, and controllable, while retaining the biocompatibility and therapeutic versatility characteristic of EV‐mediated communication.

A significant challenge in the therapeutic application of natural EVs lies in the substantial heterogeneity in molecular cargo across different cell types. The composition of EVs is greatly affected by the cellular origin, the microenvironment and the physiological or pathological state of the cells that produce them, leading to variations in protein, lipid, and nucleic acid profiles. These differences can influence their biological activity and therapeutic effectiveness [260, 261]. Although this variability allows for functional specialization and potential customization of EV‐based treatments, it also complicates the process of standardization, makes it harder to compare results across studies and challenges reproducibility, key factors for clinical application [260, 261].

The scalability of EV production presents a major challenge for their clinical development. Traditional two‐dimensional cell culture systems are inadequate to meet the extensive scale‐up requirements for therapeutic applications, and although advanced methodologies such as three‐dimensional cultures and bioreactors have shown promise in increasing EV yield, these techniques add complexity to manufacturing and quality assurance [262]. Moreover, scaling up must not compromise the integrity, composition, or bioactivity of EVs, and current bioprocess pipelines require optimization to ensure product consistency and functional properties [262].

Establishing robust, cost‐effective and reproducible large‐scale production platforms remains an unresolved challenge in the field and overcoming these obstacles will be critical to advancing EV‐based therapeutics toward clinical application [260, 262].

Regulatory agencies such as the US Food and Drug Administration (FDA) and the European Medicines Agency (EMA) pose significant challenges for the clinical implementation of EV‐based delivery systems due to the lack of specific regulatory frameworks tailored to these complex biological products. EVs share features with biologics, advanced therapy medicinal products (ATMPs) and nanoparticle drug carriers, which complicates their classification and the delineation of appropriate criteria for identity, purity, potency, and safety—fundamental requirements for regulatory approval [263].

Within the European Union, EV‐based therapeutics are often classified as ATMPs under Regulation (EC) No. 1394/2007, requiring compliance with Good Manufacturing Practice (GMP), comprehensive source material characterization, and quality assurance comparable to other advanced therapies [261, 263]. Across different regions, the lack of well‐defined, EV‐specific technical guidelines, and standardized assays for characterization and quality control remains a significant obstacle, as regulatory bodies have yet to issue harmonized guidance that clearly addresses the unique properties and heterogeneity of EV preparations [263, 264]. Therefore, establishing clear, consensus‐based regulatory frameworks that explicitly account for the distinctive features of EV products is crucial for facilitating their safe and effective clinical translation and regulatory approval. A comprehensive assessment of biodistribution, immunogenicity and long‐term safety will be necessary to meet regulatory requirements. Consequently, developing clear and harmonized regulatory guidelines is imperative to enable the successful translation of EV‐based therapeutics (Figure 2, right panel).

## 7 Clinical Translation: Current Status and Barriers

### 7.1 Landscape of EV‐Related Clinical Trials (Numbers, Phases, Disease Areas)

Over the past decade, preclinical animal models have played a central role in establishing the therapeutic potential of EVs. These studies have been crucial for understanding EV biodistribution, safety, mechanisms of action, and therapeutic efficacy, forming the foundation for current clinical trials [265, 266]. Most preclinical studies employ EVs derived from: MSCs (from bone marrow, adipose tissue, umbilical cord), neural stem cells, immune cells (e.g., DCs, macrophages), cardiac progenitor cells, and tumor cells (mainly for cancer biology and drug delivery studies) [265].

Clinical trials involving EVs for treating various human diseases represent a new frontier in modern medicine (Table 6 and Figure 2, right panel). They are particularly focused on inflammatory conditions, oncological diseases and various regenerative applications, aiming to enhance the effectiveness of traditional therapies and minimize off‐target effects [1]. Since the first clinical trial using EVs in 1999, there has been a significant rise in new clinical trials, with over 470 registered studies to date [1]. Representative examples include MSC‐derived EVs for ARDS and inflammatory lung injury, MSC‐derived exosomes for moderate COVID‐19 to ameliorate inflammation (e.g., NCT05216562), MSC‐derived exosome inhalations for post‐COVID pulmonary lesions (e.g., NCT05787288) and EVs for cancer treatment (e.g., NCT05831397). Most of these clinical trials are in Phase I and II, focusing on safety and tolerability, reflecting the early stage of clinical translation in this emerging field [1].

**TABLE 6:** List of ongoing clinical trials using EVs produced by the indicated cells (Column 4) for the treatment of the reported pathologies (Column 2).

Trial (NCT ID)	Condition/indication	Phase	EV source/type	Intervention/goal	Data source
NCT06002841	Acute respiratory failure (ARDS, COVID‐19 or other)	Phase I/II	MSC‐derived EVs	IV infusion to assess safety and efficacy vs. placebo	https://clinicaltrials.gov/study/NCT06002841?term=NCT06002841&rank=1
NCT05354141	Moderate‐to‐severe ARDS in hospitalized patients	Phase III	Bone marrow MSC‐derived EVs	IV EV therapy (ExoFlo) for ARDS treatment	https://clinicaltrials.gov/study/NCT05354141?term=NCT05354141&rank=1
NCT05787288	Post‐COVID pulmonary lesions	Early phase (I/II?)	MSC‐derived EVs	Nebulized EV inhalation for respiratory condition (safety/efficacy)	https://clinicaltrials.gov/study/NCT05787288?term=NCT05787288&rank=1
NCT05413148	Retinitis pigmentosa	Phase II/III	Umbilical cord MSC and exosomes	Assess EV therapeutic potential for retinal degeneration	https://clinicaltrials.gov/study/NCT05413148?term=NCT05413148&rank=1
NCT04173650	Dystrophic epidermolysis bullosa	Phase I/II	Allogeneic MSC‐derived EV product (AGLE‐102)	Safety and efficacy of EV treatment for skin disease	https://clinicaltrials.gov/study/NCT04173650?term=NCT04173650&rank=1
NCT06242379	Retinal/eye disease (injection)	Phase I/II	Bone marrow MSC‐derived small EVs	Intravitreal EV injection for ocular condition	https://clinicaltrials.gov/study/NCT06242379?term=NCT06242379&rank=1
NCT04493242	COVID‐19‐associated ARDS	Phase II	MSC‐derived EV infusion	Double‐blind RCT for severe COVID‐19 ARDS	https://clinicaltrials.gov/study/NCT04493242?term=NCT04493242&rank=1
NCT06431152	Knee osteoarthritis	Phase I	MSC‐derived small EVs	Intra‐articular EV therapy for OA symptoms	https://clinicaltrials.gov/study/NCT06431152?term=NCT06431152&rank=1
NCT06812637	Chronic diabetic foot ulcers (DFUs)	Pilot/exploratory	Wharton's Jelly MSC‐derived exosomes	Topical application of EVs for wound healing	https://clinicaltrials.gov/study/NCT06812637?term=NCT06812637&rank=1
NCT05475418	Adipose tissue exosome dressing	Early/pilot	Autologous adipose exosomes	Dressings with EVs for tissue repair	https://clinicaltrials.gov/study/NCT05475418?term=NCT05475418&rank=1

Table 6 summarizes some current clinical trials involving EVs that are registered or recruiting, noting that many are early phase (I–III) and use EVs derived from MSCs or other sources for therapeutic purposes (excluding diagnostic biomarker studies) [1].

### 7.2 Representative Trial Results: Successes and Failures

Preclinical studies have demonstrated that MSC‐derived EVs are the most widely used [265] and can reduce cytokine production, modulate macrophage polarization, and attenuate tissue damage. In murine models of lung injury, EV treatment improved oxygenation, reduced alveolar inflammation, and decreased mortality [265, 266]. EV administration has also led to reduced infarct size, improved cardiac function, and enhanced angiogenesis in cardiovascular models, effects largely due to EV‐associated miRNAs, proteins, and growth factors regulating apoptosis, fibrosis, and vascular remodeling [265].

In models of neurological injury, EVs can cross the BBB, reduce neuroinflammation, promote neurogenesis and improve functional recovery [266, 267]. Engineered EVs have been explored as delivery vehicles for neuroprotective drugs or RNA molecules in animal models, broadening their translational potential [265]. EVs have also promoted cartilage repair, osteogenesis and ECM remodeling, often showing comparable or superior effects to parent stem cells but with a better safety profile [265].

In cancer research, preclinical EV studies are complex and context dependent. Animal models have been used to examine tumor progression and metastasis, explore EVs as drug or siRNA delivery systems and study immune‐modulating EVs for cancer therapy. While therapeutic EVs can inhibit tumor growth in some models, tumor‐derived EVs are also known to promote cancer progression, underscoring the importance of careful EV source selection [268].

Imaging and tracking studies in animals show that EVs often accumulate in the liver, spleen, lungs, and injured tissues, with their distribution influenced by EV size, surface markers, and administration route [269]. However, questions related to long‐term safety, optimal dosing, and the effects of repeated dosing remain open research areas.

These findings have directly guided the design of early‐phase human clinical trials.

### 7.3 Major Translational Hurdles (GMP Production, Stability, Dosing, In Vivo Tracking)

Despite promising results, several major hurdles limit the clinical translation of EV‐based therapies. Preclinical studies highlight challenges related to EV biodistribution, long‐term safety, optimal dosing, and the consequences of repeated administration. Moreover, EVs show preferential accumulation in the liver, spleen, and lungs, raising questions about off‐target effects and clearance mechanisms [269].

From a manufacturing perspective, producing EVs under GMP conditions remains difficult due to the substantial variability in EV yield, purity and molecular composition. These parameters are strongly influenced by the cell source, culture conditions, and isolation method, making it challenging to achieve the level of batch‐to‐batch consistency required for clinical‐grade products [270, 271]. Ensuring EV stability during storage, transport and clinical handling represents an additional unresolved issue, as EV integrity and bioactivity can be affected by freeze–thaw cycles, buffer composition, and long‐term storage conditions. The absence of standardized protocols for EV isolation, characterization, and potency assays further complicates regulatory approval and interstudy comparability, since different laboratories often rely on nonequivalent methods that generate EV preparations with distinct properties [270].

In vivo tracking of EVs is also limited by current imaging technologies, which lack the sensitivity and resolution required to fully characterize EV pharmacokinetics, biodistribution, and clearance. This limitation makes it difficult to determine how EVs behave in the human body, how long they persist in circulation and which tissues they preferentially accumulate in. Additionally, the absence of consensus on dosing regimens, including dose units, frequency of administration and route of delivery, represents a major barrier to clinical standardization. Preclinical studies often use heterogeneous dosing strategies, making it challenging to extrapolate effective and safe dosing parameters for human trials [271].

Overall, while EV‐based therapies show strong potential, overcoming these translational challenges (ranging from GMP manufacturing and stability to in vivo tracking and dosing) will be essential for their successful integration into clinical practice.

## 8 Conclusion and Perspectives

Starting from the state of the art, future research on EVs should focus on bridging fundamental molecular insights with translational and clinical applications. A deeper understanding of the mechanisms governing selective miRNA loading in EVs is essential, especially the roles of RBPs, RNA sequence motifs, and post‐transcriptional RNA modifications. Systematic characterization of RBP–RNA interactions and their context‐dependent regulation will be critical for elucidating how specific miRNA cargoes are sorted under physiological and pathological conditions. In parallel, advances in high‐resolution omics technologies, single‐vesicle analysis, and integrated computational approaches will be necessary to map EV heterogeneity and cargo dynamics. From a therapeutic perspective, starting from basic science discoveries, future studies should focus on refining EV engineering strategies to exploit endogenous miRNA sorting pathways, enhancing cargo specificity, stability, and effective delivery. Lastly, well‐designed preclinical models and large scale, standardized clinical trials are required to validate EV‐based treatments, evaluate their long‐term safety and determine their effectiveness across various disease settings.

### 8.1 Summary of Key Concepts and Remaining Core Challenges

Despite the rapid progress in the EV field, several challenges and limitations persist. One major limitation is the incomplete understanding of the molecular rules governing miRNA selection, loading into EVs and intracellular retention. Although RBPs, RNA motifs, and RNA modifications have been found to be involved, further exploration is needed. From a translational perspective, directly modulating RBPs is generally considered a suboptimal therapeutic approach due to their pleiotropic functions and broad involvement in multiple cellular pathways. However, dissecting the epigenetic mechanisms that govern RNA‐specific RBP recruitment may reveal new and more selective therapeutic avenues. In particular, m6A RNA modification plays a crucial role in priming transcripts for RBP binding, such as hnRNPA2B1, thereby influencing RNA fate and function. Since m6A levels are often elevated in patients with oncological diseases, targeting the regulatory machinery of this modification emerges as an attractive approach. Modulating m6A levels by intervening on the methyltransferase METTL3 may therefore represent a powerful strategy to indirectly control RBP–RNA functional interactions while reducing systemic effects associated with direct RBP targeting.

From a clinical perspective, challenges include scaling up EV production, ensuring batch‐to‐batch consistency and maintaining precise control over cargo composition. Potential off‐target effects, immunogenicity and biodistribution of EVs are also critical concerns that must be carefully addressed. Moreover, current clinical trials are still limited in number and scope, making it hard to draw definitive conclusions about the therapeutic efficacy and regulatory pathways for EV‐based treatments.

### 8.2 Future Directions (AI‐Driven EV Engineering, In Vivo Imaging, Targeted Delivery)

Despite their promise in drug delivery and precision medicine, engineered nanoparticles still face substantial therapeutic limitations that restrict their clinical applicability, particularly regarding bio‐incompatibility and limited tissue specificity. Upon systemic administration, nanoparticles interact dynamically with the biological environment, leading to protein–corona formation, immune recognition, and preferential uptake by the mononuclear phagocyte system. These processes result in rapid clearance and off‐target accumulation in organs such as the liver and spleen [272], thereby reducing the effective therapeutic dose at the target site and raising concerns related to immunogenicity, cytotoxicity, and long‐term safety. Furthermore, achieving robust tissue‐ or cell‐specific targeting remains a major challenge due to physiological barriers, heterogeneous vascular permeability, and limited penetration into target tissues, all of which hinder controlled biodistribution and therapeutic efficacy [272]. Variability in nanoparticle composition, size, and surface chemistry further complicates reproducibility and translational scalability, underscoring the need for improved design strategies to enhance biocompatibility and achieve reliable tissue specificity in clinical settings or through the direct use of customized EVs [273].

Looking ahead, several emerging technological directions offer promising avenues to overcome these limitations and accelerate the development of next‐generation biologically compatible delivery systems. AI‐driven EV engineering is enabling predictive optimization of EV biogenesis, membrane composition, cargo loading, and targeting motifs by integrating machine‐learning models with high‐throughput EV profiling platforms [274]. These computational frameworks can identify structural determinants of EV tropism, guide rational surface engineering, and accelerate the design of customized EVs with enhanced therapeutic performance. In parallel, advances in in vivo imaging (including multimodal PET/MRI tracers, high‐resolution intravital microscopy, and next‐generation EV‐specific labeling chemistries) now allow real‐time visualization of EV biodistribution, trafficking dynamics, and target‐cell engagement with minimal perturbation of vesicle biology [275, 276]. These imaging innovations are essential for validating delivery efficiency, understanding EV pharmacokinetics, and informing iterative engineering cycles. Finally, increasingly sophisticated targeted delivery strategies, such as ligand‐based surface functionalization, biomimetic coatings, microenvironment‐responsive EVs, and engineered receptor–ligand pairs, are improving selective uptake by diseased tissues while minimizing off‐target accumulation [277] (Figure 2, right panel). Collectively, these future‐oriented approaches position engineered EVs as a more biocompatible, precise, and clinically scalable alternative to synthetic nanoparticles for targeted therapeutic delivery.

### 8.3 Concluding Remarks

In conclusion, EVs represent a powerful and versatile platform linking basic biomedical research to innovative therapeutic and clinical applications. The selective loading of miRNAs into EVs is a tightly regulated process, and a thorough understanding of the molecular determinants is fundamental to harnessing EVs as reliable biomarkers and as next‐generation therapeutic delivery systems.

Integrating mechanistic insights from basic research with technological advances and clinical validation will be essential to fully exploit the potential of EV‐based therapies. As understanding of RNA biology and EV biogenesis continues to grow, the rational design of EVs with tailored miRNA cargoes may open new avenues for precision medicine, ultimately improving diagnosis, prognosis and treatment of a wide range of diseases.

## Author Contributions

**Sabrina Garbo**: conceptualization, resources, formal analysis, data curation, Writing – original draft, visualization. **Francesco Marocco**: resources, formal analysis, data curation, writing – original draft, visualization. **Clemens Zwergel**: resources, data curation, writing – original draft. **Marco Tripodi**: writing – review and editing, funding acquisition, conceptualization. **Cecilia Battistelli**: writing – review and editing, funding acquisition, conceptualization. All the authors have read and approved the final manuscript.

## Ethics Statement

The authors have nothing to report.

## Conflicts of Interest

The authors declare no conflicts of interest.

## Data Availability

The data that support the findings of this study are available from the corresponding author upon request.
